# Comparative RNA-Seq analysis unfolds a complex regulatory network imparting yellow mosaic disease resistance in mungbean [*Vigna radiata* (L.) R. Wilczek]

**DOI:** 10.1371/journal.pone.0244593

**Published:** 2021-01-12

**Authors:** Uttarayan Dasgupta, Gyan Prakash Mishra, Harsh K. Dikshit, Dwijesh C. Mishra, Tejas Bosamia, Anirban Roy, Jyotika Bhati, Priti, Muraleedhar Aski, Ranjeet R. Kumar, Amit Kumar Singh, Atul Kumar, Subodh K. Sinha, Shiksha Chaurasia, Shelly Praveen, Ramakrishnan M. Nair

**Affiliations:** 1 Division of Genetics, ICAR-Indian Agricultural Research Institute, New Delhi, India; 2 Agricultural Bioinformatics, ICAR-Indian Agricultural Statistics Research Institute, New Delhi, India; 3 Crop Improvement Division, ICAR-Directorate of Groundnut Research, Junagadh, Gujarat, India; 4 Division of Plant Pathology, ICAR-Indian Agricultural Research Institute, New Delhi, India; 5 Division of Biochemistry, ICAR-Indian Agricultural Research Institute, New Delhi, India; 6 Germplasm Evaluation Division, ICAR-National Bureau of Plant Genetic Resources, New Delhi, India; 7 Division of Seed Science and Technology, ICAR-Indian Agricultural Research Institute, New Delhi, India; 8 ICAR-National Institute for Plant Biotechnology, New Delhi, India; 9 World Vegetable Center, South Asia, ICRISAT Campus Patancheru, Hyderabad, India; ICAR-Indian Institute of Agricultural Biotechnology, INDIA

## Abstract

Yellow Mosaic Disease (YMD) in mungbean [*Vigna radiata* (L.) R. Wilczek] is one of the most damaging diseases in Asia. In the northern part of India, the YMD is caused by Mungbean Yellow Mosaic India Virus (MYMIV), while in southern India this is caused by Mungbean Yellow Mosaic Virus (MYMV). The molecular mechanism of YMD resistance in mungbean remains largely unknown. In this study, RNA-seq analysis was conducted between a resistant (PMR-1) and a susceptible (Pusa Vishal) mungbean genotype under infected and control conditions to understand the regulatory network operating between mungbean-YMV. Overall, 76.8 million raw reads could be generated in different treatment combinations, while mapping rate per library to the reference genome varied from 86.78% to 93.35%. The resistance to MYMIV showed a very complicated gene network, which begins with the production of general PAMPs (pathogen-associated molecular patterns), then activation of various signaling cascades like kinases, jasmonic acid (JA) and brassinosteroid (BR), and finally the expression of specific genes (like PR-proteins, virus resistance and R-gene proteins) leading to resistance response. The function of WRKY, NAC and MYB transcription factors in imparting the resistance against MYMIV could be established. The string analysis also revealed the role of proteins involved in kinase, viral movement and phytoene synthase activity in imparting YMD resistance. A set of novel stress-related EST-SSRs are also identified from the RNA-Seq data which may be used to find the linked genes/QTLs with the YMD resistance. Also, 11 defence-related transcripts could be validated through quantitative real-time PCR analysis. The identified gene networks have led to an insight about the defence mechanism operating against MYMIV infection in mungbean which will be of immense use to manage the YMD resistance in mungbean.

## Introduction

Mungbean (*Vigna radiata* (L.) R. Wilczek) or green gram (2n = 22) is the third most important short-duration grain legume crop after chickpea and pigeon pea [1]. Mungbean is indigenous to India or Indo-Burma region, as the Central Asian region harbors the abundance of various cultivated and wild species and considered as the ‘primary center’ of genetic diversity [2]. Across Asia, mungbean is consumed as a cheap source of protein predominantly in developing countries, thus playing a remarkable role in the alleviation of protein malnutrition [3, 4]. Mungbean protein and carbohydrate are easily digestible and create less flatulence compared to other legumes and is also a good source of minerals like iron (40–70 ppm) [3, 4]. Furthermore, the relatively smaller genome size (579 mbp) also makes it like a model crop for high throughput genomic assisted breeding [5]. The mungbean cultivation is being done in a wide latitude (40° N or S), covering tropical and sub-tropical regions of the world (http://avrdc.org/intl-mungbean-network/). Globally, during 2019, mungbean was cultivated on more than 7.0 million ha area, yielding 3.5 million tonnes of grains primarily from Asian countries and is fast spreading to other parts of the world [1]. In Asia, the major mungbean producing countries include India, China, Pakistan, Bangladesh, Sri Lanka, Thailand, Myanmar, and Vietnam [6]. India is the largest producer of mungbean, with a production of 2.17 million tonnes from 4.32 million ha area. However, its average productivity is very low (~502 kg/ha) [7].

Yellow Mosaic Disease (YMD) is the most important production constraint of mungbean and other grain legumes in the Indian sub-continent. In India, the disease was first reported on the mungbean during the 1950s at the Indian Agricultural Research Institute (IARI), New Delhi [8]. Based on the resistance level of the mungbean genotype and stage of infection, the yield loss may vary between 10 to 100% [9]. Varma et al. [10] predicted that the yield loss due to YMD could be as high as $300 million in an epidemic year taking black gram, mungbean and soybean together. The disease is characterized by the appearance of bright yellow/or golden mosaic symptoms on the leaf lamina. At the initial stage, the disease starts with the appearance of small yellow specks on the veinlet of the young leaves which gradually enlarge to form irregular yellow mosaic patterns intermingled with green tissues. The severe condition leads to complete yellowing of the entire leaf lamina. The affected plants produce fewer flowers and pods with small seeds, often the flowers drop without any pod formation, thus severely reduce the grain yield [11]. YMD of legume is caused by at least seven distinct species of whitefly (*Bemisia tabaci*) transmitted ssDNA viruses belonging to the genus *Begomovirus*, family *Geminiviridae*. Amongst these seven species, mungbean is frequently infected by two species of whitefly transmitted begomoviruses, *Mungbean yellow mosaic virus* (MYMV) and *Mungbean yellow mosaic India virus* (MYMIV). Both these species contain a bipartite genome and have a very narrow host range within the legumes. MYMV is most prevalent in Western and Southern India, Thailand and Indonesia, while MYMIV is present in Central, Eastern and Northern India, Pakistan, Bangladesh, Nepal and Vietnam [3, 12]. Recombination between these two species though does not occur frequently but some reports indicated the occurrence of recombinant viruses in northern India [13]. Different isolates of MYMIV from northern India have been fully characterized from different legumes [11].

Due to the non-availability of diverse resistant sources to YMD, most of the commercially available cultivars were derived from a few resistant sources [12, 14], which has resulted in a narrow genetic base, especially for the YMD resistance. Long-term improvement of mungbean production needs uncovering of the molecular mechanisms of YMD resistance and its use for realizing the potential yield.

To get insights into the molecular mechanism leading to resistance and susceptibility, it is essential to characterize the differential gene expression resulting due to host-virus interactions. Although, RNA-Seq analysis has been carried out in black gram and soybean under MYMV and MYMIV infection, respectively [15, 16]; however, limited efforts have been made to dissect the molecular basis of YMD resistance in mungbean through the RNA-Seq approach. The RNA-Seq analysis of MYMIV resistant and susceptible mungbean genotypes under infected and control conditions will provide a deeper understanding of the physiological changes and adjustments that occur during compatible and incompatible interactions. Thus, the study intends to identify the transcriptomic scaffolds associated with differential physiological adjustments in resistant and susceptible genotypes during YMV infection which result in the establishment of incompatible reactions in the resistant genotype.

## Materials and methods

### Plant material and virus inoculation

A yellow mosaic disease resistant mungbean genotype, PMR-1 (a selection from KM-14-44) and a susceptible genotype, Pusa Vishal were used for the RNA-Seq analysis. PMR-1 showed field resistance in initial screening during 2015–16 under field condition and was subsequently reconfirmed for its resistance in fields during 2016–17, 2017–18 and 2018–2019. The susceptible genotype, Pusa Vishal was released during the year 2000 for spring-summer cultivation in the North-Western Plain Zones (NWPZ) of India, however, in due course of time, this showed susceptibility to MYMIV infection [13] (S1 File).

The seeds of both the genotypes were sown in plastic pots (three seeds per pot) of size 15 cm (diameter) containing growing media consisting of coco peat: vermiculite: sand in 1:2:1 ratio under the partially controlled glasshouse conditions of the National Phytotron Facility at IARI, New Delhi. The growing conditions were maintained at 21°C (day) to 18°C (night) with 14:10 h as light:dark period with 70–80% humidity and plants were watered at 3–4 days interval. The viruliferous whiteflies collected from the infected mungbean plants were used for the inoculation of both resistant and susceptible genotypes under controlled conditions using standard procedures [14, 17]. The infection was confirmed through MYMIV and MYMV CP gene-specific PCR amplification and sequencing of the product (Details are included in S1 File).

Seven days following the inoculation, the apical leaves were cut and immersed in the RNAlater solution (Sigma) and then stored at −80°C. Four sample combinations viz. resistant inoculated (MRI), resistant control (MRC), susceptible inoculated (MSI) and susceptible control (MSC), each with three biological replications were pooled to increase the detection accuracy of RNA-Seq analysis.

### RNA extraction, cDNA library preparation and sequencing

Frozen leaves samples stored in the RNAlater were used for the isolation of total RNA using RNA plant mini kit (Qiagen, Hilden, Germany). The quality was checked on 1% agarose gel and NanoDrop (Thermo Fisher Scientific Inc.), while its integrity was measured using Agilent 2100 Bioanalyzer (Agilent Technologies, Palo Alto, CA, USA). The total RNA (1.0 μg) having 260/280 values between 2.0–2.1, 260/230 between 2.0–2.3 and RIN (RNA integrity number) above 7.0 were used for the cDNA library preparation using NEBNext® UltraTM RNA Library Prep Kit for Illumina®. The Illumina HiSeq 2500 sequencer (Illumina Inc., USA) was used for sequencing of qualified cDNA libraries, where both the ends of the inserts were sequenced at Nucleome Informatics Private Limited, Hyderabad, India.

### Sequence pre-processing, rRNA removal, read alignment, novel transcript identification and calculation of their abundance

To find the quality of raw sequences, FastQC (www.bioinformatics.babraham.ac.uk/projects/fastqc); while Adapter Removal-v2 (V2.2.0) and our in-house scripts were used for trimming of the adapter sequences, low-quality bases (Phred score <30) and short sequences (<50 bp). The high-quality reads (HQRs) were aligned with Silva database using Bowtie2 (V2.2.9), rRNA sequences were removed and reads were aligned to the assembly of *Vigna radiata* (http://plantgenomics.snu.ac.kr) using TopHat (V2.0.13) and BAM (Binary Alignment Map) format was used to generate the transcript annotations as Gene Transfer File (GTF) format using Cufflinks (V2.2.1). The ‘StringTie’ was used to find all the potential transcripts and validation was performed against the reference *Vigna radiata’s* GTF file using ‘gffcompare’, and ‘Subread’ was used to analyze the gene expression levels.

### Differential gene expression analysis

After aligning the HQRs with mungbean reference genome, the read counts of each gene were normalized by calculating the fragments per kilobase of exon per million fragments mapped (FPKM). The transcript expression and differential expression were estimated by cufflinks (ver. 2.2.1) and Cuffdiff program of cufflinks package, respectively. The genes were considered significantly expressed in each of the four sample comparisons if they had a log2-fold change (log2FC) ≥2 (up-regulated) or ≤-2 (down-regulated) with an FDR/p-adj of 0.01. MA and volcano plots are used to infer the overall distribution of DEGs, while edgeR was used at a dispersion parameter of 0.1. The transcripts were functionally classified and represented as a heat map using Multi-experiment Viewer (MeV v4.9.0), while the the number of transcripts among various comparisons was plotted as Venn diagram using Vennplex [18]. Gene ontology (GO) enrichment and pathway analysis of transcripts.

The annotation data of transcripts from all the four comparisons were used for the identification of GO terms using topGO, an R-bioconductor package for enrichment analysis. The enrichment of identified GO terms was performed through PlantRegMap (http://plantregmap.cbi.pku.edu.cn) [19] at P<0.01. The Kyoto Encyclopedia of Genes and Genomes (KEGG) Automatic Annotation Server (KAAS) was used for the ortholog assignment and mapping of the CDs to the biological pathways. Further, clusterProfiler R-bioconductor package was used for the pathway enrichment at a p-value Cutoff of 0.05.

### Protein-protein interaction (PPI) network analysis

To understand the PPI network of the DEGs, String network analysis was performed. The selected gene lists of resistant and susceptible plants were first uploaded into the string database (https://string-db.org/) [20] and then MCODE [21] plugin of Cytoscape [22] was performed for the identification of highly interconnected proteins. The predicted protein-protein interaction networks were based on the sequence similarity of the legume species *Glycine max*.

### PHI-Base analysis

PHI-Base analysis of the DEGs has been performed using online tool PHI-base 4 (www.phi-base.org) to find the cross-kingdom comparative network approaches for pathogenicity, virulence and effector genes discovery and also to identify potential biotic stress intervention targets during MYMIV-mungbean infection [23].

### Validation of the expression profiles through qRT-PCR

To compare the RNA-Seq data with qRT-PCR data, the leaf tissues from both, control and treated genotypes were taken in three replications at 07-day post-inoculation from the same set of disease induction experiment as previously explained. Eighteen DEGs are randomly chosen from RNA-Seq data for qPCR validation. The cDNA of each replication and each sample preparation was diluted (1:10) using nuclease-free water, primers were designed using batch primer3 [24] and the reaction was performed in 20 μL mixture containing 10μL of 2×SYBR^®^ Green ROX qPCR FAST mastermix (QIAGEN, USA), 1μL of cDNA template, 20 pM of each primer, and final volume was maintained using nuclease-free water. The qPCR was carried out in 48-well blocks StepOne™ System (Applied Biosystems) and the amplification was performed (10 min at 95°C, followed by 40 cycles of 95°C for 15s and 60°C for 30s). To normalize the variance among samples, actin was used as endogenous control and Livak’s 2^-ΔΔCT^ method [25] was used for the analysis.

### Development of stress associated genic-SSR markers and polymorphism assessment

The RNA-Seq data of all four samples were used for the mining of stress associated genic-SSRs. To do away with the SSR primers already available in the public domain, the candidate sequences were searched and removed from our RNA Seq data using an in-house Perl script [26]. The novel genic SSR markers were identified from the remaining transcripts using MIcroSAtellite (MISA) tool [27], and primers were designed using online tool BatchPrimer3 v1.0 [24] using set parameters [26]. Thirty-nine primer-pairs were synthesized (Europhin Genomics, India) and subjected to polymorphism measurement in a panel of 42 diverse mungbean genotypes (S1 Table) which are routinely used in mungbean improvement breeding program. The PCR amplified products were resolved on 3% Metaphor agarose gel (Lonza, Rockland, USA) and documented using a gel documentation system (Alpha Imager).

## Results

### RNA-Seq read mapping and statistics

The Illumina sequencing for four libraries from both resistant (PMR-1) and susceptible (Pusa Vishal) genotypes under infected (Resistant Infected: RI, Susceptible Infected: SI) and control (Resistant Control: RC, Susceptible Control: SC) conditions generated a total of 76.8 million raw reads, ranging from 17.4 to 20.7 million reads per library with an average GC content of 43–44%. These reads were subsequently passed through several quality filters and after removing the low complexity reads, adaptor, primer sequences and rRNA sequence, ~73.2 million (95.36%) HQRs having ≥30 Phred value have been obtained (Table 1). All the raw data were deposited in the National Centre for Biotechnology Information (NCBI) Sequence Read Archive (SRA, http://www.ncbi.nlm.nih.gov/sra) with the accession number PRJNA631469. The HQRs were then aligned to the genome of *V*. *radiata* using TopHat tool and when mapped, showed a range of 86.78% to 93.35% of the reads per library (S2 Table).

**Table 1:** Raw and clean sequence details of resistant and susceptible mungbean genotypes under virus challenged and control conditions.

Sample	Total Sequences		Sequence length		GC (%)	
	Value (Raw)	Value (Clean)	Value (Raw)	Value (Clean)	Value (Raw)	Value (Clean)
MRI	183,68,774	176,06,377 (95.85%)	150	26–140	43	43
MSI	207,58,708	197,91,535 (95.34%)	150	26–140	44	44
MRC	202,44,230	192,67,306 (95.17%)	150	26–140	44	44
MSC	174,27,015	165,70,600 (95.09%)	150	26–140	44	44
**Total**	**767,98,727**	**732,35,818 (95.36%)**	**-**	**-**	**-**	**-**

Where, MRI: Mungbean Resistant inoculated, MSI: Mungbean Susceptible inoculated; MRC: Mungbean Resistant control and MSC: Mungbean Susceptible control.

### Identification of Differentially Expressed Genes (DEGs)

**Fig 1:**
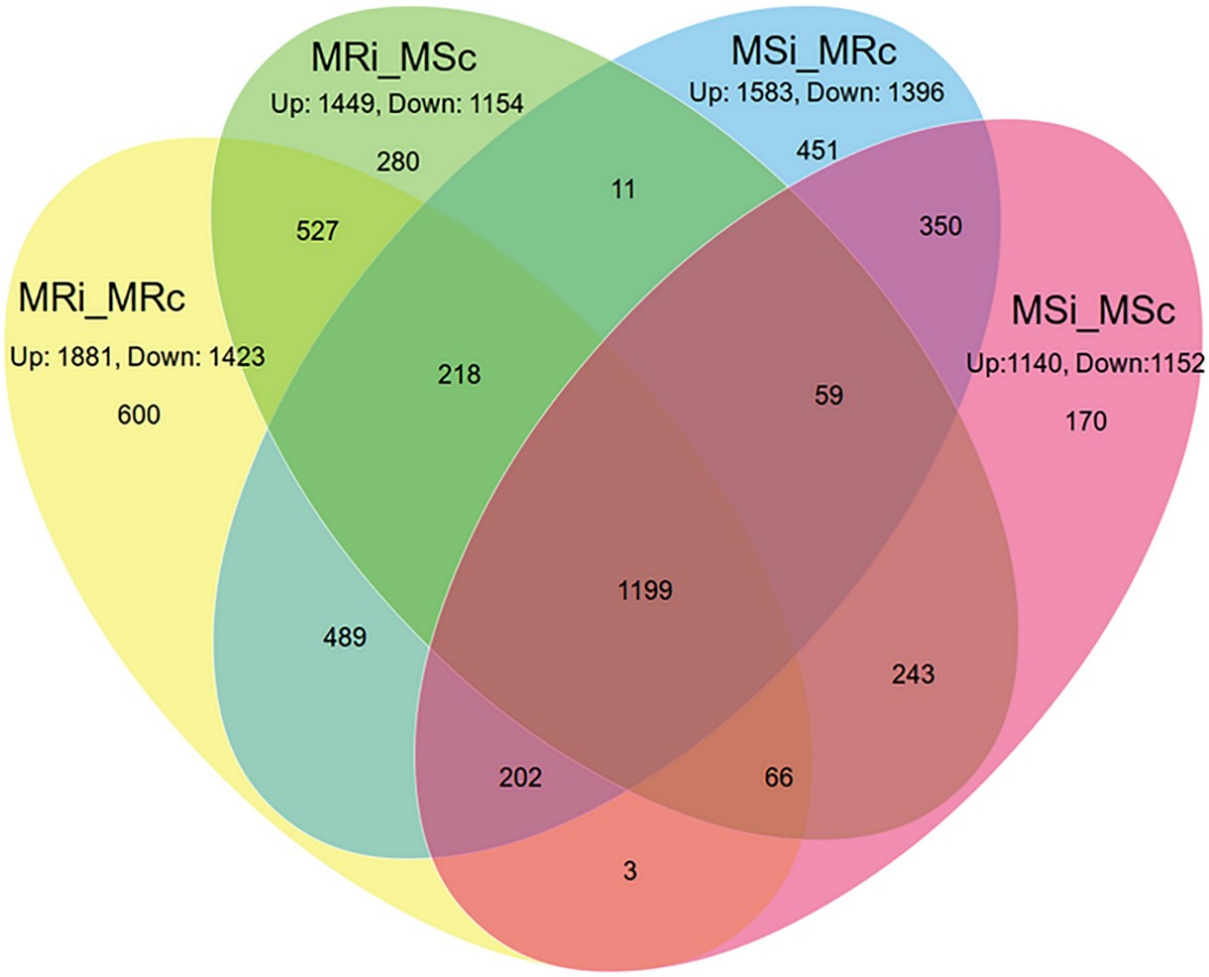
Venn diagram representing the proportion of up-regulated and down-regulated genes in different combinations. Where MRi: Mungbean Resistant Inoculated; MRc: Mungbean Resistant Control; MSi: Mungbean Susceptible Inoculated; MSc: Mungbean Susceptible Control.

The DEGs were identified between resistant (PMR-1) and susceptible (Pusa Vishal) genotypes under MYMIV infected and control conditions. Detailed four-way comparisons of each sample were performed with *V*. *radiata* genome, to find the number of up-regulated and down-regulated transcripts at adjusted p≤0.05 and log2≤-2.0 (S3–S6 Tables). The DEGs analysis upon changing the threshold to log3≥3 and log3≤-3 revealed 94, 75, 60, and 39 genes as significantly up-regulated; while 96, 71, 155 and 104 genes as significantly down-regulated in MRI_MRC, MRI_MSC, MSI_MRC, and MSI_MSC combinations, respectively (S7 Table). However, the number of significant DEGs were decreased drastically when the threshold was changed to log4≥4 and log4≤-4 (S7 Table). The common DEGs in various experimental combinations were visualized as Venn diagram (Fig 1; S1 Fig) in the four groups which were further divided into 15 subgroups. Volcano plots and MA plots were also used to visualize the DEGs in different combinations (S2 Fig).

The majority of the DEGs got induced upon YMV infection and the detailed mapping on the *V*. *radiata* genome revealed 1881, 1449, 1583 and 1140 genes as up-regulated while, 1423, 1154, 1396 and 1152 genes as down-regulated in MRI_MRC, MRI_MSC, MSI_MRC and MSI_MSC combinations, respectively (Fig 1; S3–S6 Tables). Overall, 1501 unique transcripts have been identified of which 12.32% (600/4868), 5.75% (280/4868), 9.26% (451/4868) and 3.49% (170/4868) were found belonging to MRI_MRC, MRI_MSC, MSI_MRC and MSI_MSC combinations, respectively.

**Fig 2:**
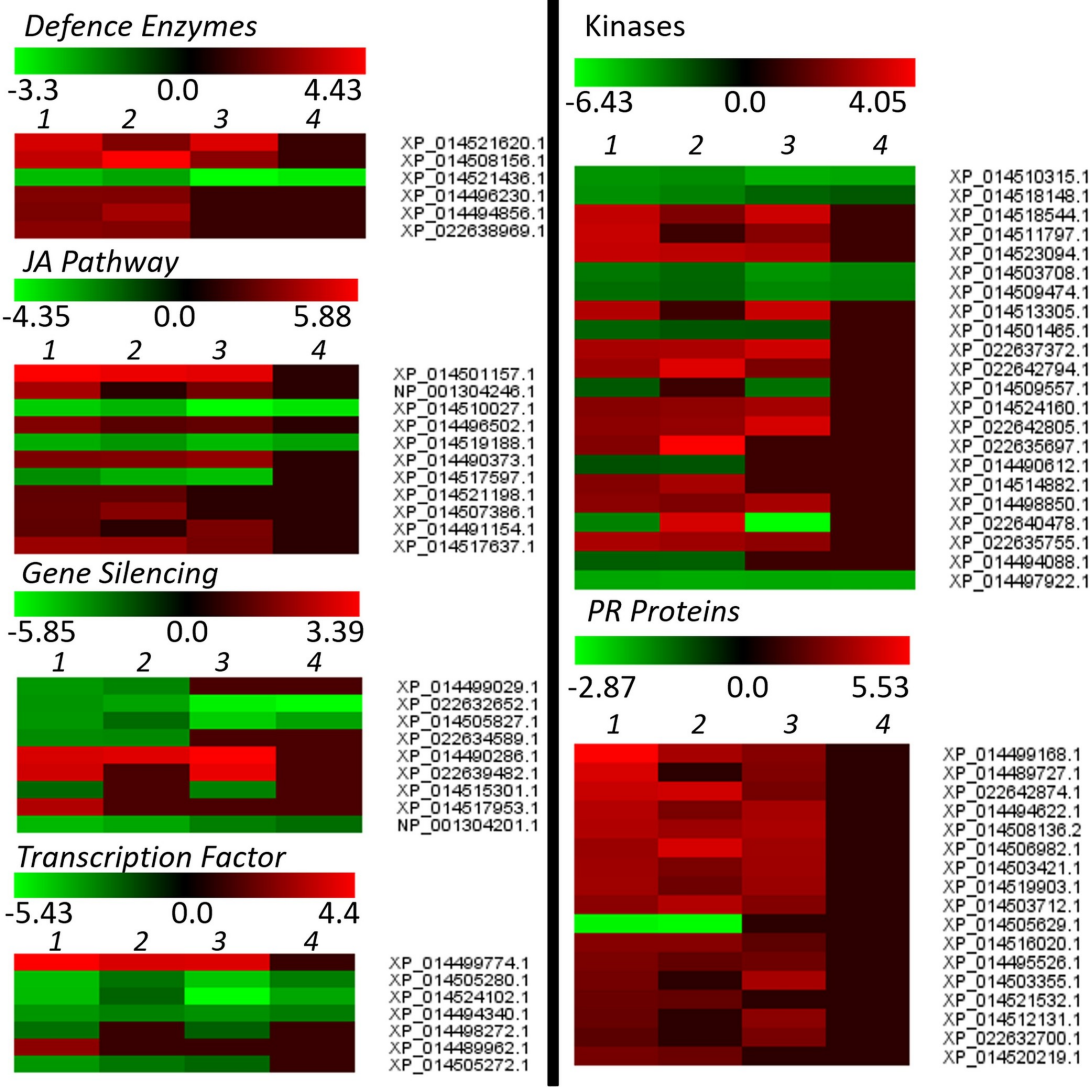
Heat map generated using stress-specific DEG groups like defence enzymes, JA pathway, gene-silencing, transcription factor, kinases and PR proteins.

The PMR-1 specific DEGs or the DEGs with more drastic expression in PMR-1 than in Pusa Vishal seems to play a key role in regulating cellular defense responses against MYMIV infection. To identify the important genes and the gene-network imparting YMD resistance in PMR-1, we then extracted a subset of biotic stress-related DEGs like defence enzymes, JA pathway, gene silencing, transcription factor, kinases and PR proteins and heat maps were generated (Fig 2, S8 Table).

Upon YMV infection, various classes of anti-oxidative enzymes, peroxidase, (S)-2-hydroxy-acid oxidase and classes of lipoxygenase were found up-regulated, while proteins that enhance the production of free radicals *viz*. oxidoreductase, 2OG-Fe(II) oxygenase family protein, pheophorbide an oxygenase, ribulose bisphosphate carboxylase oxygenase activase, chlorophyllide a oxygenase were found down-regulated in resistant genotype. In addition, there are other defence responsive enzymes such as 4-coumarate:CoA ligase like, O-methyltransferase, arogenate dehydratase 1, D-3-phosphoglycerate dehydrogenase showed up-regulation in resistant (Inoculated) sample when compared to resistant (Control) and susceptible samples. The expressions of cell wall development associated enzymes (like cellulose synthase, pectinesterase, chitinase, and lignin biosynthesis) were found up-regulated, while plant stature related genes (e.g. gibberellin and auxin biosynthetic enzymes) were found both positively and negatively regulated in MYMIV-infected mungbean plants.

Also, some other major pathways involved in the imparting MYMIV resistance include hormone-regulated immune signaling, brassinosteroid, and plant-pathogen interaction pathways, while some key defence imparting proteins include defensin-like protein domain-containing proteins, phenylpropanoids, etc. are thoroughly discussed in the following section.

### GO and KEGG analyses for the functional enrichment of *V*. *radiata* DEGs in response to MYMIV infection

**Fig 3:**
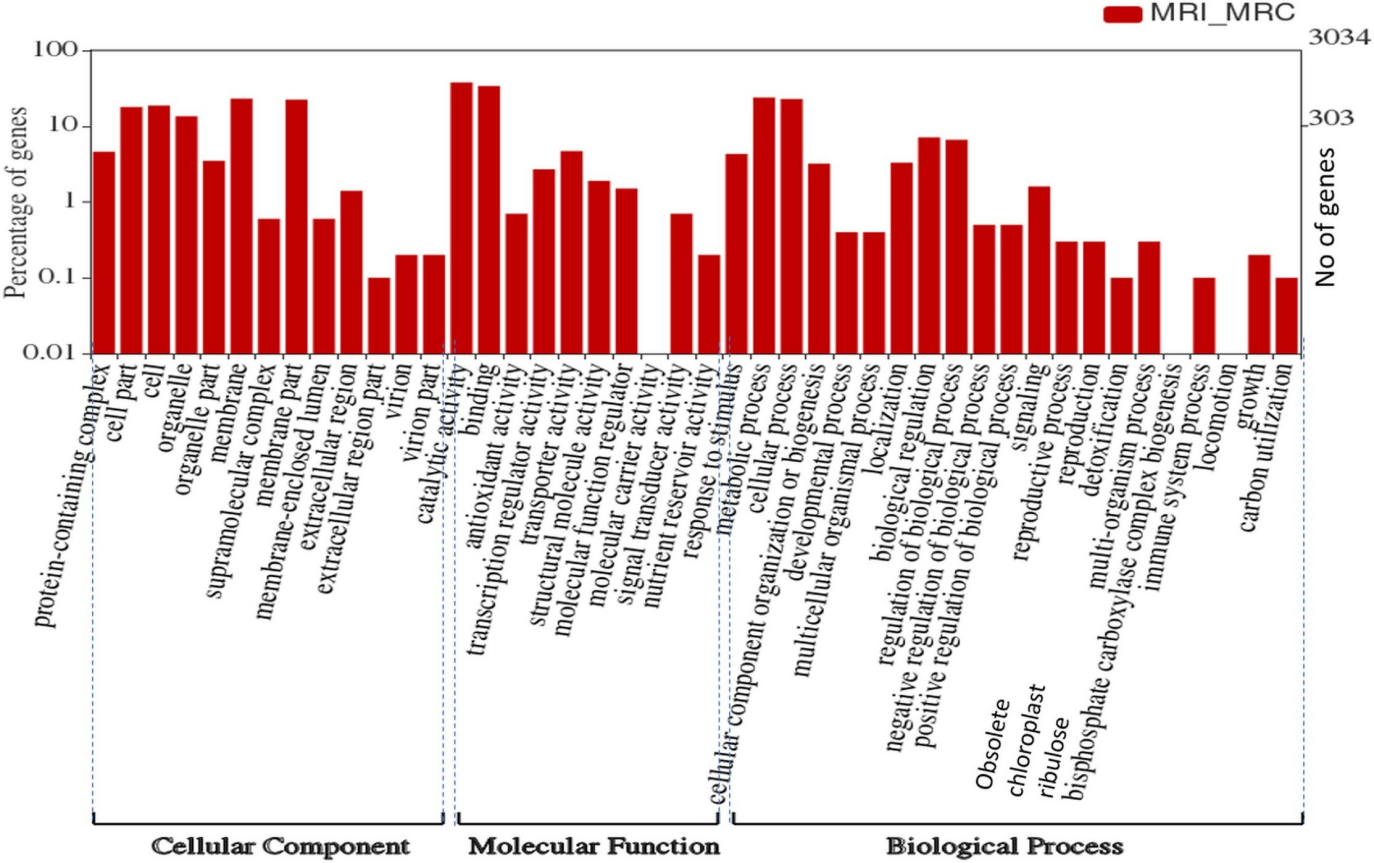
Gene ontology (GO) categorization of the differentially expressed genes (DEGs) in mungbean resistant infected (MRI) vs resistant control (MRC) combinations. There are three significantly enriched categories of biological process (BP), cellular component (CC) and molecular function (MF). Where, Y-axis represents unigene percentage.

To understand the associated biological processes which respond to the MYMIV infection in mungbean, GO analysis was performed which could identify three GO terms viz. cellular component, biological process and molecular function which are significantly over-represented for up- and down-regulated core DEGs (Fig 3, S3 Fig; S3–S6 Tables). As shown in Fig 3, most of the enriched GO terms for up-regulated core DEGs belonged to the different categories of biological processes, like signaling, carbohydrate metabolism, pigment biosynthesis, microtubule, cell wall biogenesis and photosynthesis. For cellular component, the enriched GO terms are for membrane, virion parts, nucleus, intracellular, cell-wall, nucleosome, chloroplast, mitochondrion, and MCM complex.

Accordingly, GO terms related to molecular functions, such as antioxidant, signal transduction, transcription and transporter activity, microtubule binding, hydrolase and chlorophyll binding activities were found overrepresented in MRI_MRC (S3 Table). Also, various disease-associated GO terms identified include protein kinase, hydrolase, catechol oxidase, phosphorelay sensor kinase, and oxidoreductase activities.

**Fig 4:**
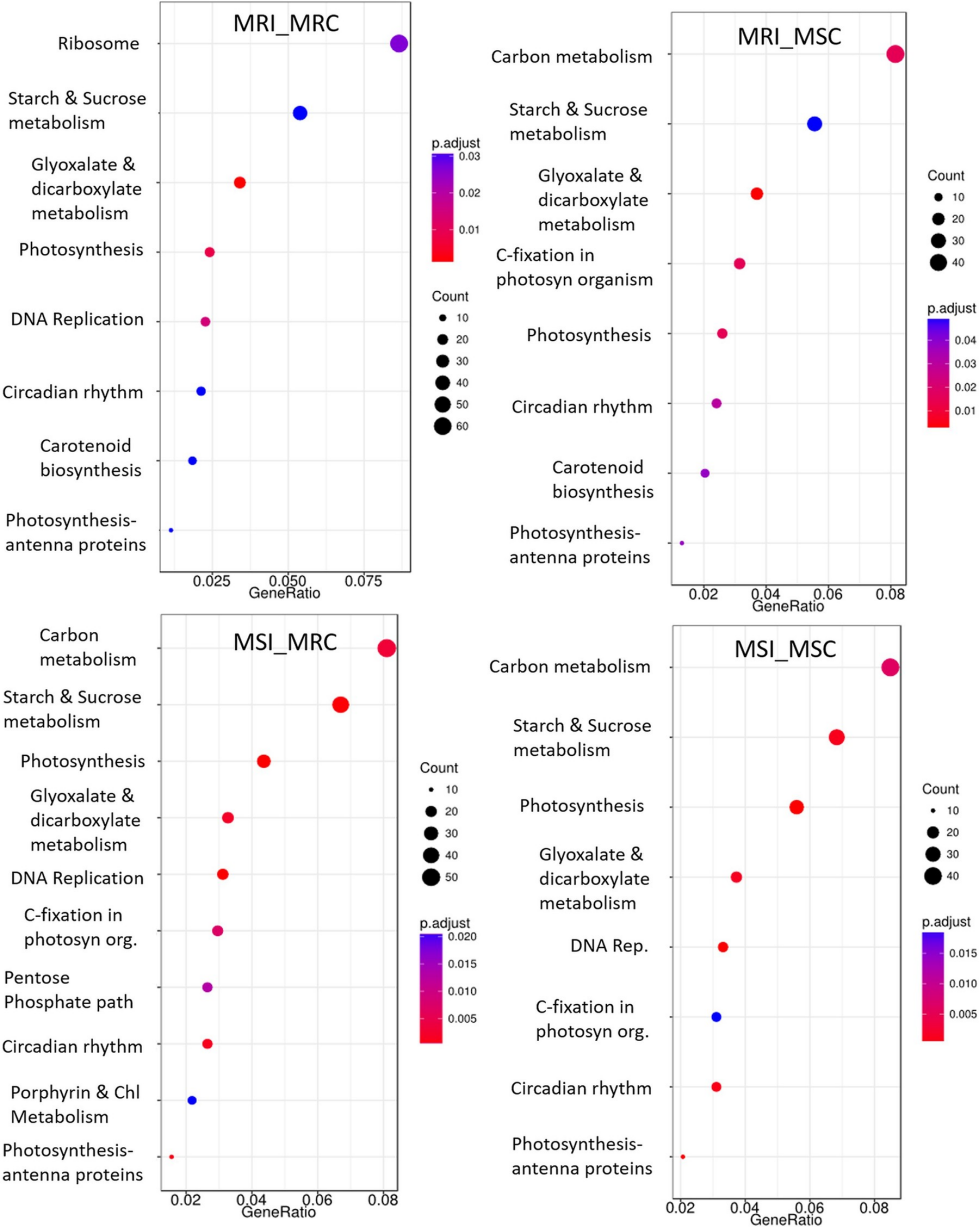
**KEGG pathways of key significantly enriched DGEs in different comparisons (a-d) representing the degree of enriched DGEs in a pathway.** Where, gene-ratio is the ratio of input-genes to the total gene-list in the pathway; the number of enriched DGEs in any pathway is indicated by the circle area, and circle color represents the range of corrected p-value.

To comprehend the major metabolic processes altered during MYMIV infection, core DEGs were mapped to KEGG pathways database and the over-represented pathways include ‘DNA replication’, ‘photosynthesis’, ‘starch and sucrose metabolism’, ‘glyoxalate and dicarboxylate metabolism’ and ‘carbon metabolism’ (Fig 4). It is interesting to note that except in the MRI_MRC combination we did not find the representation of ‘ribosome’ in any other comparison, while ‘carbon metabolism’ was dominantly represented in all the comparison except MRI_MRC.

### Protein-protein interaction (PPI) network

**Fig 5:**
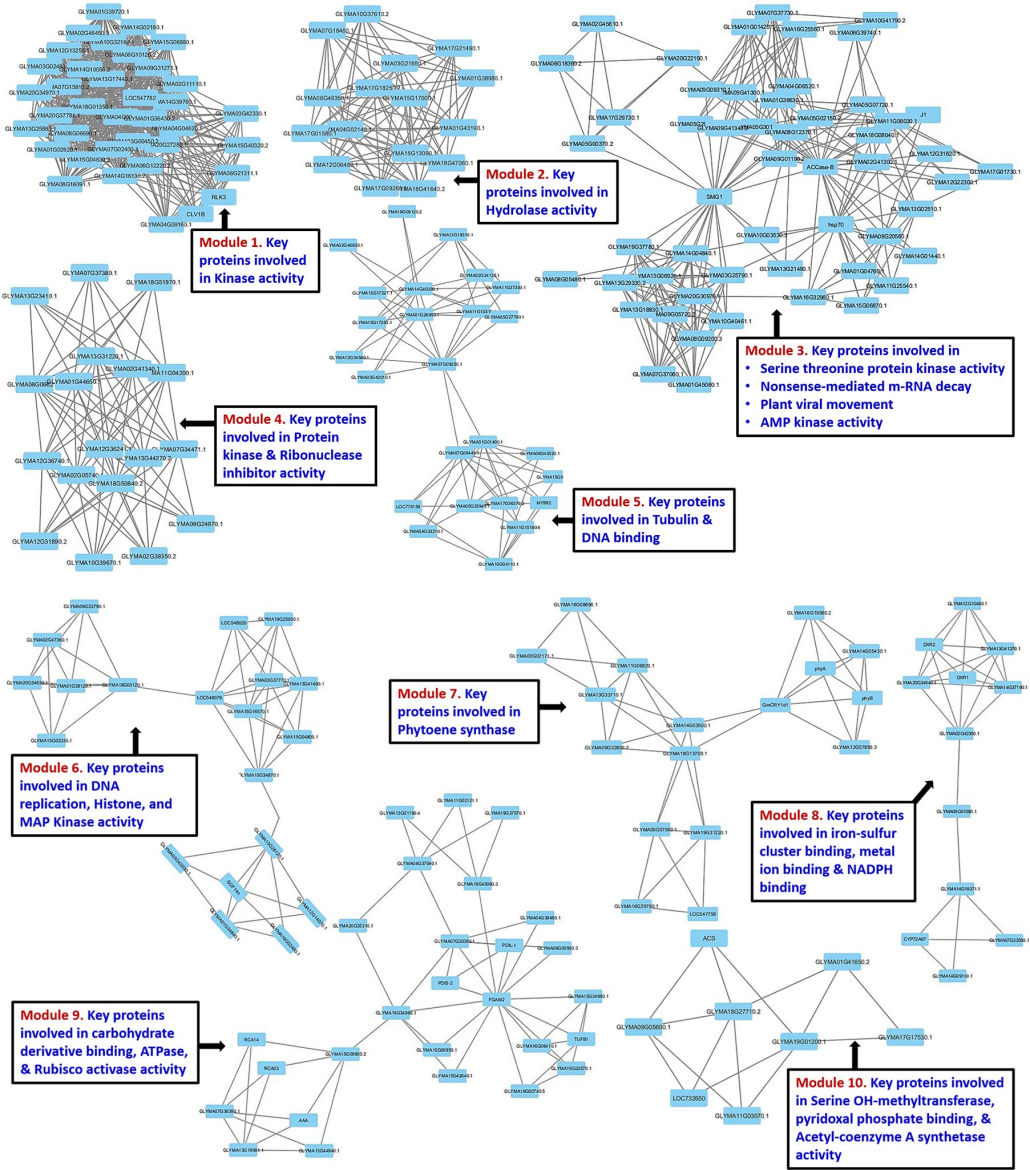
Protein-protein interaction network of (a-b) resistant genotype using MCODE analysis.

A graphical clustering software MCODE was used to find the tightly connected regions in large PPI networks representing molecular complexes in both resistant and susceptible mungbean genotypic combinations. The predicted protein-protein interaction network of resistant plants was divided in 10 major functional modules corresponding to respective pathways (Fig 5A and 5B). Module-1 and 2 consists of proteins involved in kinase and the hydrolase activity, respectively. Whereas, module-3 contains the proteins involved in non-sense mediated m-RNA decay (NMD) and virus movement; while module-4 and 5 found having ribonuclease inhibitor and DNA binding proteins, respectively. Module-6 consisted of DNA replication and histone biosynthesis proteins; whereas module-7 possessed phytoene synthase proteins which result in the biosynthesis of carotenoids. The module-8 contained metal-ion binding activity proteins, while module-9 and 10 have the proteins with the ATPase and Acetyl Coenzyme A synthetase activities, respectively.

However, the PPI analysis in susceptible genotypic combination resulted in five major functional modules (S4 Fig); where module 1 and 2 consisted of DNA binding and aminotransferase activity proteins, respectively. Whereas, module 3 and 4 contain proteins related to the pectin-lysis and DNA confirmation, respectively, while module-5 possessed alpha-amylase and calcium ion binding activity proteins. PPI network was also validated through STRING database, which revealed nearly similar interactions for both resistant (S5 Fig) and susceptible (S6 Fig) interactions.

### PHI-Base analysis of the DEGs

PHI-base analysis has been performed to find the molecular and biological information on the key genes affecting MYMIV-mungbean interactions including the information on the target sites of some anti-infective chemistry. The PhiBase hits have identified 13,730 transcripts as gene homologues that are associated in pathogen-host interactions, which include both compatible and incompatible reactions. These genes are further classified into five major categories *viz*. reduced virulence (40.23%), unaffected pathogenicity (20.67%), loss of pathogenicity (13.15%), effector (9.77%), increased virulence (6.54%) and miscellaneous (9.47%) (Table 2).

**Table 2:** PHiBase analysis of DEGs identified in response to MYMIV infection in mungbean.

Categories	Count	Percentage
Reduced_Virulence	5523	40.23
Unaffected_Pathogenicity	2838	20.67
Loss_of_Pathogenicity	1805	13.15
Effector_(Plant_Avirulence_Determinant)	1341	9.77
Miscellaneous	1300	9.47
Increased_Virulence_(Hypervirulence)	898	6.54
Resistance_To_Chemical	25	0.18
Total	13730	100

### Quantitative real-time qRT-PCR expression analysis

**Fig 6:**
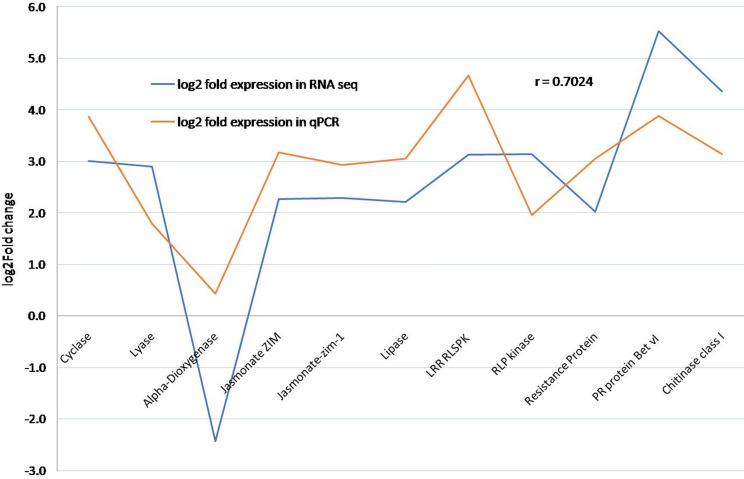
Correlation analysis between RNA-Seq and qPCR data of 11 differentially expressed transcripts.

Based on the functional classification of defence related transcripts, 18 genes were selected for the qRT-PCR based validation, while the *actin* gene was used as an endogenous control (Table 3). At seventh day of infection, the expression of Chitinase increased by four-fold in MRI when compared to MSI; whereas in susceptible genotype, its expression got suppressed. A similar pattern of expression was observed in Receptor-like kinase (RLK), Jasmonate ZIM domain-containing protein, Hydroperoxide Lyase and PR protein Bet vI family. Further comparison of these 11 differentially expressed transcripts as identified from RNA-Seq with that of qPCR data revealed a positive correlation (r = 0.7024) (Fig 6).

**Table 3:** Details of RT-PCR primers used for the validation purpose.

S. No.	Seq ID	Gene	Forward primer (5’-3’)	Tm (°C)	Reverse primer (5'-3')	Tm (°C)	Size (bp)	log2 fold expression (RNA-Seq)	log2 fold expression (qPCR)
1	NP_001304246.1	Lipoxygenase	CCACCGTTGACACTCTCACTT	60.20	TTTCCCACCTTTCCTTTTCC	60.27	100	3.880	Not Amplified
2	XP_014510027.1	Glyoxalase	TCCAAATCCCGACCCTTT	60.25	CAAGGATTGCCTTCAGCTTT	59.45	101	-3.527	Multiple Tm peak
3	XP_014496502.1	Allene oxide cyclase	CCCTTTGTAGCCACCACTTT	59.10	TGTGCTGAGGGTGTTTTGAG	59.87	110	3.006	3.873
4	XP_014519188.1	Triacylglycerol Lipase	TGAGGAAAATGTGGGAAGAGA	59.66	TTGGAAAATCCACCAAGACC	59.77	113	-2.984	Multiple Tm peak
5	XP_014490373.1	Hydroperoxide Lyase	ATCAAGCAACAAGGGAAGGA	59.67	GGAATTGAACCCTAGCACGA	60.07	105	2.895	1.793
6	XP_014517597.1	Alpha-Dioxygenase	GGCAGAAATATGCTCCCTGT	59.15	TGAATTGCTTCCCTGTTTCC	60.05	115	-2.433	0.427
7	XP_014521198.1	Jasmonate ZIM domain-containing protein	ATCCCTACCACTGCCAACAA	60.38	CCAAGAACCGATGAAGTGAAG	59.72	109	2.265	3.171
8	XP_014507386.1	Jasmonate-zim-domain protein 1	TCTGAAGCATCGTGTCAACC	59.84	TAAGCAGGCTCCGAAGAGAA	60.23	104	2.292	2.934
9	XP_014491154.1	Triacylglycerol Lipase	CAGATGCTCGTGATGTTGAGA	60.00	CCATTACATAGTCGGCGTGAG	60.53	108	2.205	3.046
10	XP_014517637.1	Glyoxalase/ Dioxygenase superfamily	CAGTTGTGGAAGAAGGTGGAA	60.13	GGAAATTGGAAGCACAGGAA	60.05	110	3.581	Multiple Tm peak
11	XP_014518544.1	LRR receptor-like serinethreonine-protein kinase	TTAGCAGCTCCCTTCCAAAA	59.95	TGTGAGCAGACCGGATAGAA	59.39	104	3.128	4.668
12	XP_014523094.1	Receptor like protein kinase	GGCTTTCTGATGAAGGGAAG	58.86	TCATGGAGTGTGCAGAGGAG	59.98	118	3.140	1.951
13	XP_022642794.1	MAP kinase	AGCCGAAGTTGAAGGTGAAA	59.85	GGTAGACTGTGCCGAAGGAG	59.87	107	2.489	Not Amplified
14	XP_022642938.1	Resistance Protein	TTAGGGGGACGCAGATAACA	60.46	GATCTCCTTCGGCAACACA	59.78	107	2.803	Not Amplified
15	XP_014521820.2	Resistance Protein	TGAAGCCTCCTCTTCCTCCT	60.47	GTTCCCACTTTGCTGTTTCC	59.57	110	2.020	3.049
16	XP_014499168.1	PR protein Bet v I family	TGAAGGCCACACGAAAATAC	58.62	ATCAACAGCCTTGGCAAACA	61.61	104	5.527	3.877
17	XP_022642874.1	Chitinase class I	ATGGACCAGCAGGACAGAAC	60.12	AGGGCGAACAAACTCCATC	60.07	119	4.354	3.145
18	XP_014495526.1	Cysteine-rich repeat secretory protein	ACAGTGCGAGGGTAATTTGG	59.99	CCTGCGCGGAAATAGAGTAG	60.00	101	2.707	Multiple Tm peak

### Development of novel stress associated EST-SSR markers from the *Vigna radiata*-MYMIV transcriptome

To find the novel non-redundant SSR markers from RNA-Seq data, all publicly available SSR primers of mungbean were searched and positive sequences were removed [26]. In total, 2468 novel SSR motif could be identified of which, mono-nucleotide (46.3%) was most abundant followed by di-nucleotide (26.1%) and tri-nucleotide (25.9%) motifs. However, tetra-nucleotide (1.2%) penta-nucleotide (0.3%) and hexa-nucleotide (0.2%) motifs was least frequent (Table 4). The total sequence length examined using MISA was 129,74,744 bp which possessed 2468 SSRs having total length of 4537 bp. Additionally, the relative abundance (SSR per Mb) and relative density (bp/Mb) of SSR was recorded as 190.22 and 349.68 bp/Mb, respectively. Overall, 514 novel primer pairs (S9 Table) could be designed and a set of 39 primers were randomly selected (from all the chromosomes) for the PCR validation on a panel of 42 diverse mungbean genotypes (S1 Table). Of these, 36 primer-pairs could be amplified, while 10 displayed the polymorphism. The number of alleles detected and PIC value of polymorphic primers were ranged from 1–3 and 0.1–0.79, respectively (S1 Table).

**Table 4:** *V*. *radiata* SSR and motif details as obtained from MISA analysis.

Motif	% (Number)	Length [bp]	Longest motifs	Repeat abundance % (Number)
Mono:	46.3% (1142)	1142	A: 32, T: 50, C: 13, G: 11	A/T: 99.7% (1139); C/G: 0.3% (3)
Di:	26.1% (644)	1288	43: AT	AC/ GT: 5.6% (36), AG/ CT: 60.2% (388), AT/ AT: 34.2% (220)
Tri:	25.9% (640)	1920	17: TAT	AAC/ GTT: 10.6% (68), AAG/ CTT: 27.2% (174), AAT/ ATT: 14.1% (90), ACC/ GGT: 12.0% (77), ACG/ CGT: 2.3% (15), ACT/ AGT: 2.2% (14), AGC/ CTG: 8.4% (54), AGG/ CCT: 6.7% (43), ATC/ ATG: 15.0% (96), CCG/ CGG: 1.4% (9)
Tetra:	1.2% (29)	116	6: ACTC, ATGC, ATTA, CATC, CTTT, GAGT, TCAC, TTAT	AAAC/ GTTT: 6.9% (2), AAAG/ CTTT: 31.0% (9), AAAT/ ATTT: 13.8% (4), AACC/ GGTT: 3.4% (1), AAGG/ CCTT: 3.4% (1), AATT/ AATT: 6.9% (2), ACTC/ AGTG: 17.2% (5), ATCC/ ATGG: 13.8% (4), ATGC/ ATGC: 3.4% (1)
Penta:	0.3% (7)	35	5: AAGTG, ACAGA, CTCTT, GTTGT, TGATA	AACAC/ GTGTT: 42.9% (3), AACAG/ CTGTT: 14.3% (1), AAGAG/ CTCTT: 14.3% (1), AAGTG/ACTTC: 14.3% (1), ATATC/ ATATG: 14.3% (1)
Hexa:	0.2% (6)	36	7: CCGTCG	AAAGAG/ CTCTTT: 16.7% (1), AACCCC/ GGGGTT: 33.3% (2), ACGGCG/ CCGTCG: 33.3% (2), ATCCCC/ ATGGGG: 16.7% (1)
**Total**	**2468**	**4537**	**-**	**-**

## Discussion

YMD is one of the most important diseases of mungbean in which the resistance mechanism at the molecular level is still unknown. Thus, the RNA-Seq approach has been attempted using resistant and susceptible genotypes to unravel the possible resistance mechanism involving the formation of PAMPs, activation of various signaling cascades, resulting in the expression of specific resistance imparting genes causing YMD resistance response against this begomovirus. The details of resistance mechanism functioning against MYMIV infection have been thoroughly explored and discussed. Overall, 76.8 million raw reads could be generated in different treatment combinations, while Liu et al. [28] have performed RNA-Seq analysis on 38.3–39.8 million reads. The mapping rate per library to mungbean reference genome varied from 86.78% to 93.35% which was much higher than that of 61.78 to 75.87% as reported for blackgram when studied under MYMIV infection [29]. Among the four combinations studied, maximum transcripts were found differentially regulated in resistant than the susceptible mungbean genotype which was similar to the RNA-Seq analysis of blackgram infected with MYMIV, indicating some association between the degree of altered gene expression and resistance expression [30].

The interaction between geminivirus encoded proteins with the host protein kinases, suggesting their role in altering various defence associated signal transduction pathways [31, 32]. The receptor-like kinase (RLKs), conferring immunity are PRRs (pattern-recognition receptors) which are associated with the host cell membranes and could identify PAMPs (pathogen-associated molecular patterns) of the virus by binding with elicitors and instigate an array of defence related signal cascades or PTI (PAMP triggered immunity) and thereby impart resistance [33]. Thus, the up-regulation of RLKs in the resistant mungbean genotype appears crucial for the YMV infection by triggering the downstream defence responses involving complex signaling cascades [34]. Also, several other kinases like mitogen-activated protein kinases (MAPK), serine threonine-protein kinase, lectin protein kinase, tyrosine-protein kinase etc. were found differentially expressed having their role as a signaling molecule for the upstream activation of immune responses against YMV in mungbean plants [35], while serine/threonine kinases are found most abundant.

The downstream activation of PTI and effector-triggered immunity (ETI) also result in the activation of varied hormone-regulated immune signaling pathways [36]. Jasmonic acid (JA) is one of the key defence hormones which is reported inhibiting photosynthesis and cell division, and thereby maintaining the balance between growth and defense in plants [37, 38]. The jasmonate-ZIM-domain (JAZ) proteins and lipoxygenase (LOX) proteins were found up-regulated in resistant inoculated over susceptible inoculated combinations. A JAZ protein resembling domain in the PPD2 (plant-specific putative DNA-binding *proteins*) is known as a host factor for begomoviruses which bind to the DNA elements in the viral CP promoter region and thereby regulating the virus multiplication [39]. Besides, NAC genes by acting on the MYC2 gene can also regulate the JA-signaled defense responses [40]. The LOX pathway products viz., colneleic and colnelenic acids when accumulated in the leaves of TMV infected potato plants cause inhibitory activity [41]. The transcriptome data also showed differential expression of 3-epi-6-deoxocathasterone 23-monooxygenase, a BR (brassinosteroid) synthesis-related gene upon MYMIV infection, as also reported by Li et al. [42] in *Nicotiana*. Thus, complex networks of genes appear regulating the MYMIV defence response in mungbean.

Several pathogenesis-related TFs and ‘plant-pathogen interaction’ pathways genes such as WRKY, bHLH, NAC, Myb family, a calcium-binding protein, and respiratory burst oxidase homolog protein were found altered for mungbean, maize [31] and interspecific derivative of mungbean and ricebean [43]. After infection, the virus maneuvers the host cell cycle mostly by the deregulation of cell cycle checkpoints, and the key host genes with altered expression include systemic acquired resistance (SAR), cell cycle, WRKY and NAC TFs [44, 45]. WRKY function in signaling during virus recognition and thereby activation of defence mechanisms [46]. Various WRKY-TF were also found induced in cucumber after CCYV (cucurbit chlorotic yellows virus) infection [47].

Likewise, *NAC* transcription factors can also impart defense against virus infections through signal transduction affecting viral DNA replication [48]. The geminivirus replication initiator protein *Rep* by stimulating both viral and plant DNA replication interferes with the host DNA replication [49]. The MYMIV Rep, *w*heat dwarf geminivirus (WDV) *RepA* and tomato leaf curl virus (TLCV) replication enhancer *REn* proteins were found interacting with NAC transcription factor [50–52]. Interestingly, geminivirus *Rep*-interacting motor protein (XP_014492749.1) was found down-regulated in the resistant genotype and appears to contribute towards the YMD resistance response in mungbean.

Up-regulation of pathogenesis-related (PR) proteins, defensin-like proteins, and other LRR domain-containing proteins like GTP-binding proteins have been known to be associated with the induction of defense-related genes [44, 53]. The MYB gene action gets modulated by JA, and it functions by activating the PR genes expression at the site of infection by triggering the SAR and thereby protecting the plant against viral infection [54, 55]. Likewise, in tomato, the overexpression of OsMYB4 reported protecting the plants against viral infection [56]. A range of PR proteins like PR protein Bet vI family, PR protein 5-like are found highly up-regulated in mungbean. Other DEGs in this category include cysteine-rich repeat secretory protein, defensin-like protein and Plant lipoxygenase, etc.

When effectors of pathogen overcome PTI, plants use their resistance (R) proteins to activate another type of immune response called effector-triggered immunity (ETI) wherein R-proteins identifies the effectors as *Avr* (avirulence) factors, which often results in a hypersensitive response (HR) as resistance mechanism [57]. Thus, upon infection, the virus was recognized by plant R-genes which lead to the downstream synthesis of a range of R-proteins, anti-oxidative enzymes, disease responsive proteins and secondary metabolites leading to the resistance response [58]. The defence enzymes or resistance-related genes identified included glutathione S-transferase, heat shock protein (HSPs), and ferredoxin as also identified by Zhou et al. [31].

During infection, the genes associated with secondary metabolites production also gets activated [59] and flavonoids and isoflavonoid production genes showed upregulation in resistant mungbean sample. The repression of photosynthesis-related genes has been reported in response to the virus infection [60, 61] which appears as a yellow mosaic symptom in the mungbean. Except for chlorophyllase-2, the expression of all of the chlorophyll- and carotenoid-related genes expressed a decreased trend. Also, the expression of the cytochrome P450 gene was found significantly affected upon YMV infection [31].

Phenylpropanoids play important roles in plant responses towards various stresses and phenylalanine ammonia-lyase (PAL) contributes to several pathways including phenylpropanoid biosynthesis and its expression was found upregulated in response to CCYV (cucurbit chlorotic yellows virus) infection in *Cucumis sativus* [47]. Correspondingly, the proteins involved in the repression of phenylpropanoid biosynthesis were also found down-regulated in our study.

RNA silencing can impart antiviral defense to the plants through the involvement of multiple factors like endoribonuclease dicer, RNA binding, RNA polymerase, RDR, AGO and Heat Shock cognate 70 [57], which showed differential expression in our study too. Upon YMV infection, conflict starts between plant and virus, and plant encoded Argonaute (AGO) family proteins help in the suppression of this conflict [62]. Besides, RNA-dependent RNA polymerase (RDR6) also confers antiviral activities in plants [63].

The endochitinases are known to get induced upon pathogen attack and can be correlated with the host resistance [64]. Similar results were also observed for maize in response to the virus infection [31] and several cell wall development associated enzymes were found differentially regulated in our study too. Protein degradation pathways involving ubiquitin-proteasome system which was reported contributing to the RBSDV resistance in maize [31], was also found responding to the YMV infection in mungbean.

**Fig 7:**
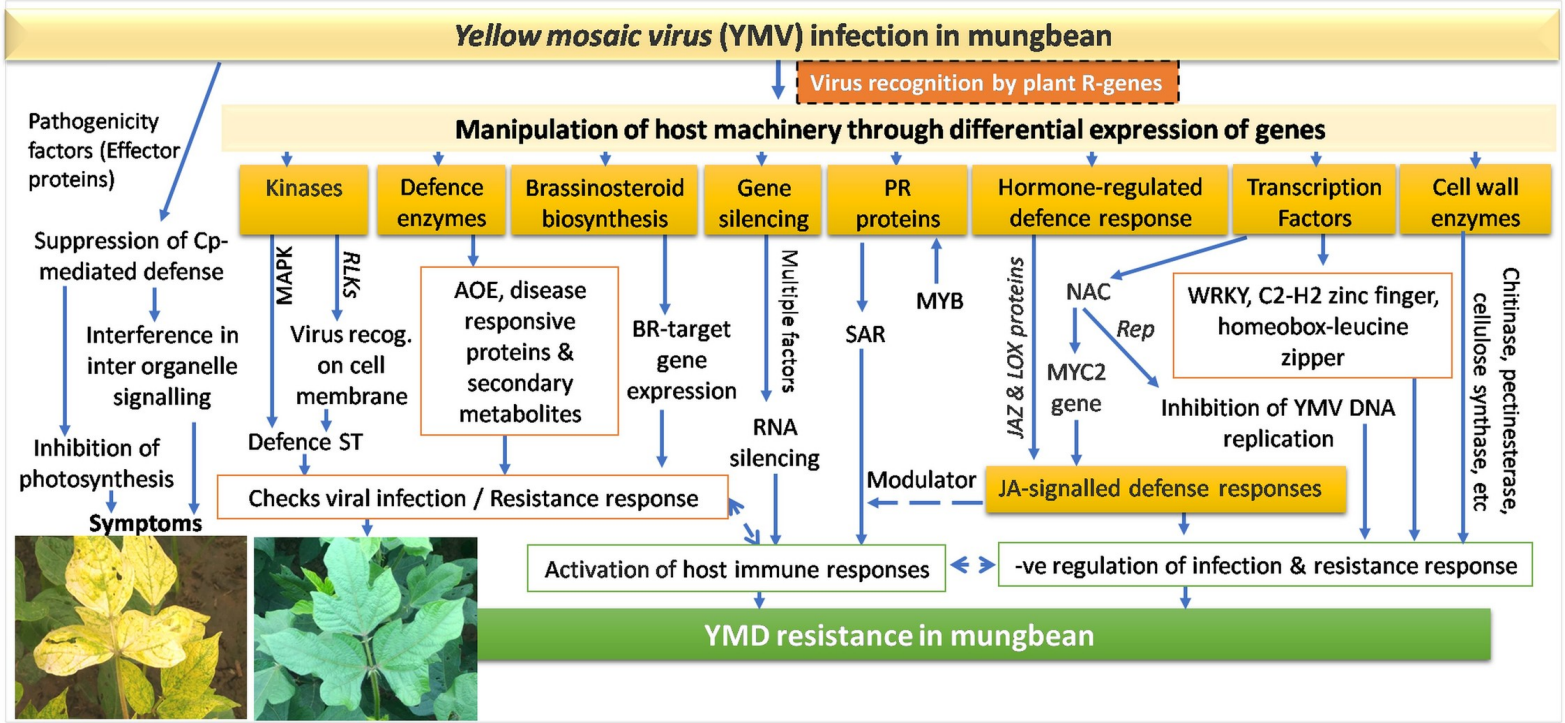
The complex gene network giving an insight into the probable defence mechanism leading to YMD resistance in mungbean.

Recently, a major QTL (qMYMV4_1) on chromosome number 4 of mungbean is reported harboring 16 disease resistance candidate genes, including serine/threonine-protein kinase superfamily (STK), MYB–TF, WRKY family–TF, zinc finger, RING/FYVE/PHD-type protein, small GTP-binding protein, RLKs, bHLH TF, DEAD/DEAH box hydrolase, JA carboxyl methyltransferase (JMT) and cytochrome P450 (CYP) genes. Similarly, CYP, DNA/RNA helicase, DEAD/DEAH box type protein, zinc finger and RING/FYVE/PHD-type proteins were reported as part of the plant defence response network to the viral infection in cucumber [65]. Interestingly, we could also identify the differential expression of all these R gene families and the TF as the candidate gene(s) having their role in imparting YMD resistance in mungbean too. Notably, we could not identify any NBS-LRR transcript as also reported by Mathivathana et al. [43]. Thus, based on the function of several key genes that are identified associated with the imposition of YMD resistance in mungbean, a comprehensive gene network has been worked out as Fig 7.

All the DEGs with GO allotted (4030) across all samples were subjected to gene enrichment analysis and 96 GO terms got enriched. The enriched GO terms are further visualized as a bubble plot (S7 Fig). Among various GO terms, 'response to ethylene' (GO:0009723) and 'response to jasmonic acid’ (GO:0009753) are significantly enriched which supported the hypothesis of operation of JA/ET mediated resistance pathway during mungbean-MYMIV interaction.

Despite numerous common GO terms between MYMIV resistant and susceptible mungbean genotypes, considerable heterogeneity has been recorded in the identified DEG pool as also reported for urdbean [30] with the major representation of 'Starch and Sucrose Metabolism' in the resistant while 'photosynthesis' in the susceptible combination. As reported by Li et al. [42], we also observed significant enrichments of the photosynthesis, photosynthesis antenna proteins and carotenoid biosynthesis pathway in the KEGG pathway analysis of YMV-infected plants. The photosynthesis-related genes are reportedly affected in several virus infections including RSV (Rice Stripe Virus) [66] and AMV (Alfalfa Mosaic Virus) [67] as the chloroplast plays an active role in inter-organelle signaling and imposition of defense response and virus try to suppress the chloroplast-based defense through effectors [68].

The MCODE analysis has helped in fine-tuning of PPI clusters in both resistant and susceptible mungbean genotypes. The PPI network for the YMD resistant genotype has identified the proteins primarily involved in the kinase, hydrolase and ribonuclease inhibitor activities imparting resistance especially through NMD, HSP70, ACCase, phytoene synthase and metal ion binding activity. Of these, the NMD functions by degrading the mRNAs having premature termination codon, while HSP70 has its role in the intracellular movement of plant virus. Further, ACCase is the rate-limiting enzyme in the fatty-acid biosynthesis which gets inhibited by AMP kinase and thus limits the viral replication. We could also identify the proteins involved in the synthesis of phytoene synthase (Psy) as also reported by Ibdah et al. [69] for PepMV infection in tomato. The 'Iron-Sulfur Cluster (ISC) assembly protein' is required for the maturation of RNase L Inhibitor 1 (RLI1) proteins, which in infected cells forms various RNA–protein complexes such as ribosomal particles, translation initiation complexes, and virus particles [70]. Thus, the down-regulation of certain ISC protein in resistant mungbean genotypes seems to help in the prevention of virus maturation.

In addition, the heat shock protein 70 (HSP70) is known to be required for the synthesis of viral genome [71, 72] and in the present investigation this was found down-regulated in the both resistant and susceptible genotypes however the magnitude of down-regulation is much higher in resistant (-4.25) over susceptible (-2.51) genotype. In plants, the inositol-requiring proteins and bZIP transcription factor is known to play important role in protein response signaling network and in the absence of bZIP60 reduced level of TuMV accumulation was recorded [73]. On a similar note, we could also found down-regulation of bZIP71 in both resistant and susceptible genotype with different magnitude.

An adequate number of polymorphic molecular markers is the prerequisite for the success of any molecular breeding program [74, 75] and our study identified 7688 novel SSR motifs with tri- and di-nucleotide repeats as most abundant [26, 76]. The primer validation using 39 stress-specific EST-SSR primers on a set of 42 genotypes varying for YMD resistance revealed 92% amplification, while 26% revealed polymorphism which was in tune with previous reports [26, 77] suggesting the usefulness of these novel markers in molecular breeding aiming for stress tolerance in mungbean.

## Conclusions

As observed in many plants, the resistance to MYMIV in mungbean was found involving a very complicated gene network, which begins with the production of general PAMPs, then activation of various kinase-signaling cascades, and finally an expression of specific genes (like PR-proteins, defense-related proteins, antioxidative enzymes and secondary metabolites) leading to resistance response in mungbean [78] (Fig 7). The function of WRKY, NAC and MYB transcription factor, RLKs, cytochrome P450, JAZ and LOX genes in imparting the resistance against MYMIV could be established which will provide a detailed understanding of the YMD resistance mechanisms in mungbean. Down-regulation of genes coding for receptor-like protein kinase, resistance protein and PR protein Bet v I family through CRISPER/Cas9 or RNAi technology in the resistant genotype may be attempted to find its effect on plants reaction for the resistance response. Additionally, information about the genes causing incompatible interactions with YMVs and the novel EST-SSRs (especially located on chromosome 4) will improve our ability to fine map the linked resistance genes for its ultimate transfer to the commercial varieties [43, 79]. The complex, massive and coordinated change in the gene network which has been unveiled in this study has not only led to an insight about the defence mechanism against MYMIV infection but also presented a novel perspective about the gene networks leading to YMD resistance in mungbean.

## Supporting information

S1 Fig Venn diagram representing the proportion of up-regulated and down-regulated genes in different combinations (at log3≥3 and log3≤-3). Where MRI: Mungbean Resistant Inoculated; MRC: Mungbean Resistant Control; MSI: Mungbean Susceptible Inoculated; MSC: Mungbean Susceptible Control.
(TIF)

S2 Fig MA plots and Volcano plots of DEGs in (a). MRI_MRC, (b). MRI_MSC, (c). MSI_MRC, (d). MSI_MSC combinations. The fold change in the gene expression was shown by the abscissa while the adjusted p-values for the differential expression were represented by the vertical coordinates. Genes with no-significant differences are indicated by black dots while red dots represent the differentially-regulated genes.
(TIF)

S3 Fig Gene ontology (GO) categorization of the differentially expressed genes (DEGs) in *V*. *radiata* in (a) Resistant Infected (MRI) vs Susceptible Control (MSC), (b) Susceptible Infected (MSI) vs Resistant Control (MRC), (c) Susceptible Infected (MSI) vs Susceptible Control (MSC), combinations. There are three main categories of biological process, cellular component and molecular function. Where Y-axis represents the unigene percentage.
(TIF)

S4 Fig Protein-protein interaction network of susceptible genotype using MCODE analysis. (TIF)

S5 Fig Interaction network constituted through STRING analysis for YMD resistance interactions. (TIF)

S6 Fig Interaction network constituted through STRING analysis for YMD susceptibility interactions. (TIF)

S7 Fig Visualization of GO enriched terms (during mungbean-MYMIV interaction) as a bubble plot. (TIF)

S1 File (PDF)

S1 Table (XLS)

S2 Table Alignment details of total paired and unpaired reads. (DOCX)

S3 Table Details of the DEGs as obtained from the MRI vs MRC sample comparision. (XLS)

S4 Table Details of the DEGs obtained from the MRI vs MSc sample comparision. (XLSX)

S5 Table Details of the DEGs as obtained from MSI and MRC sample comparision. (XLS)

S6 Table Details of the DEGs as obtained from MSI vs MSC sample comparision. (XLS)

S7 Table Number of DEGs at threshold of log to the base 3 and 4. (XLSX)

S8 Table List of stress related genes selected for heat map formation. (XLSX)

S9 Table Details of the novel stress specific EST-SSR primers designed from the RNA Seq data. (XLS)
