# Supplementary figures and images for: Comparative RNA-Seq analysis unfolds a complex regulatory network imparting yellow mosaic disease resistance in mungbean [Vigna radiata (L.) R. Wilczek]
Source: PLoS One. 2021 Jan 12;16(1):e0244593. doi: 10.1371/journal.pone.0244593 (PMC7802970; doi:10.1371/journal.pone.0244593)

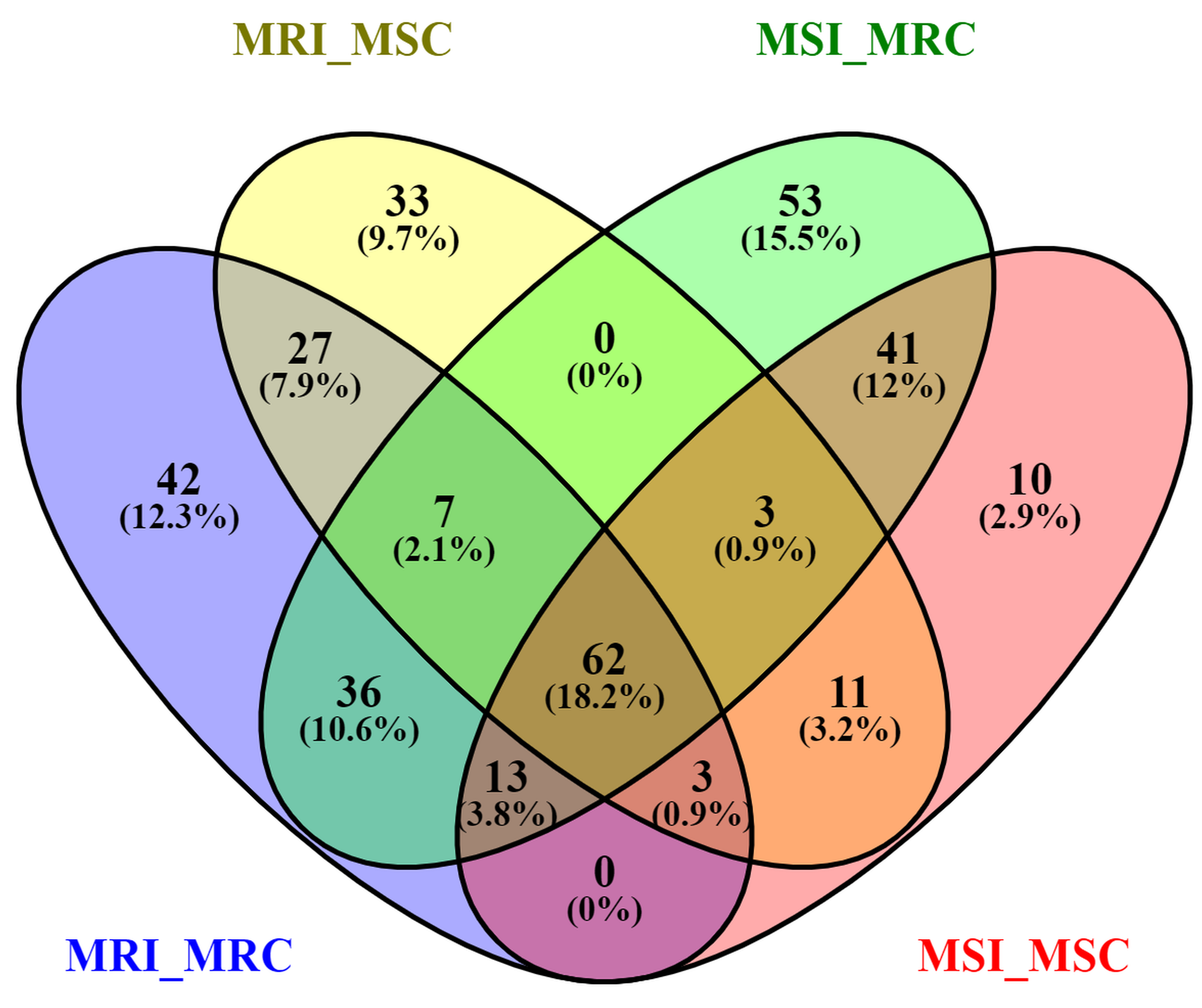

Supplement: S1 Fig — Where MRI: Mungbean Resistant Inoculated; MRC: Mungbean Resistant Control; MSI: Mungbean Susceptible Inoculated; MSC: Mungbean Susceptible Control. (TIF) [file pone.0244593.s001.tif]

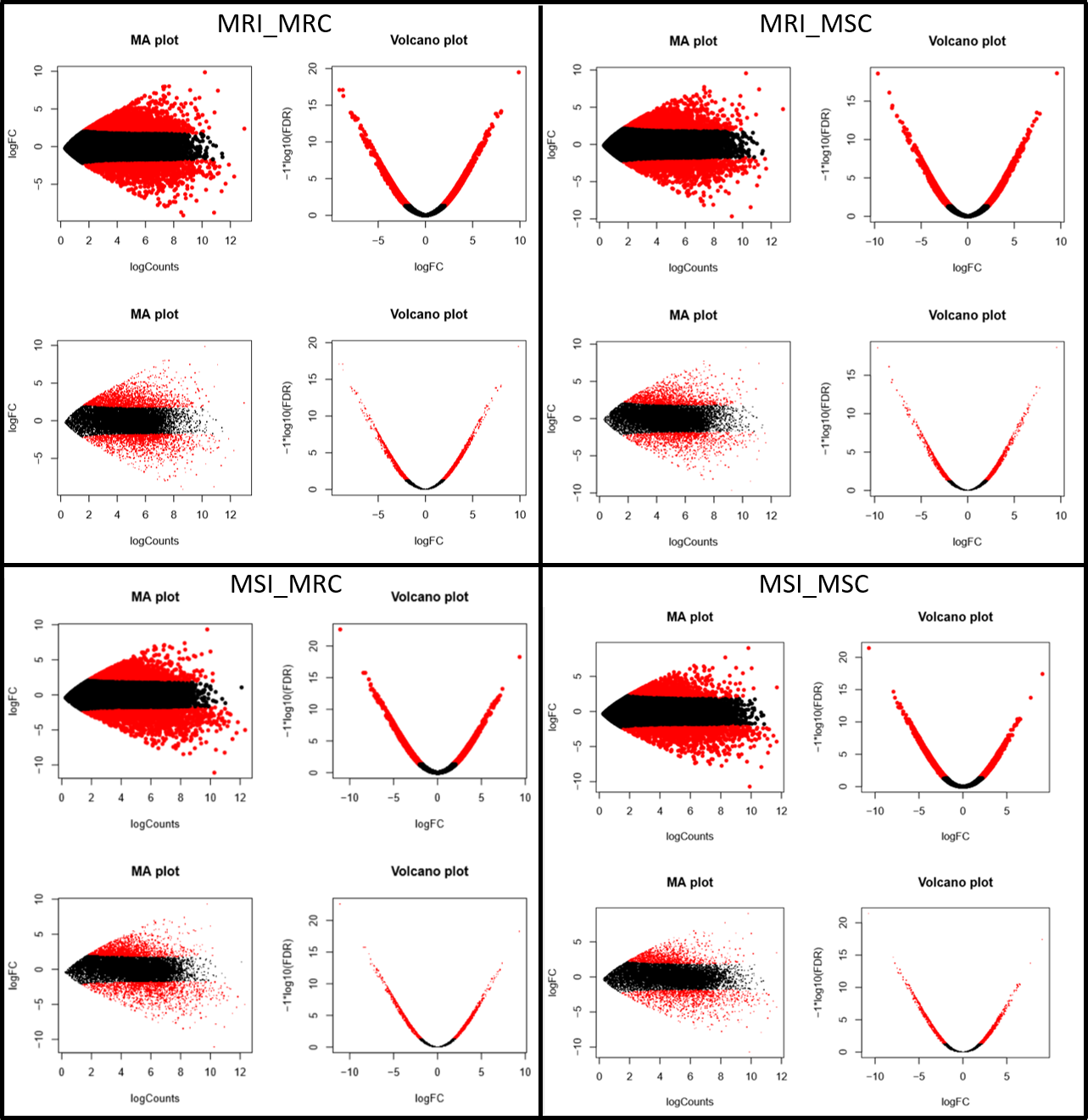

Supplement: S2 Fig — MA plots and Volcano plots of DEGs in (a). MRI_MRC, (b). MRI_MSC, (c). MSI_MRC, (d). MSI_MSC combinations. The fold change in the gene expression was shown by the abscissa while the adjusted p-values for the differential expression were represented by the vertical coordinates. Genes with no-significant differences are indicated by black dots while red dots represent the differentially-regulated genes. (TIF) [file pone.0244593.s002.tif]

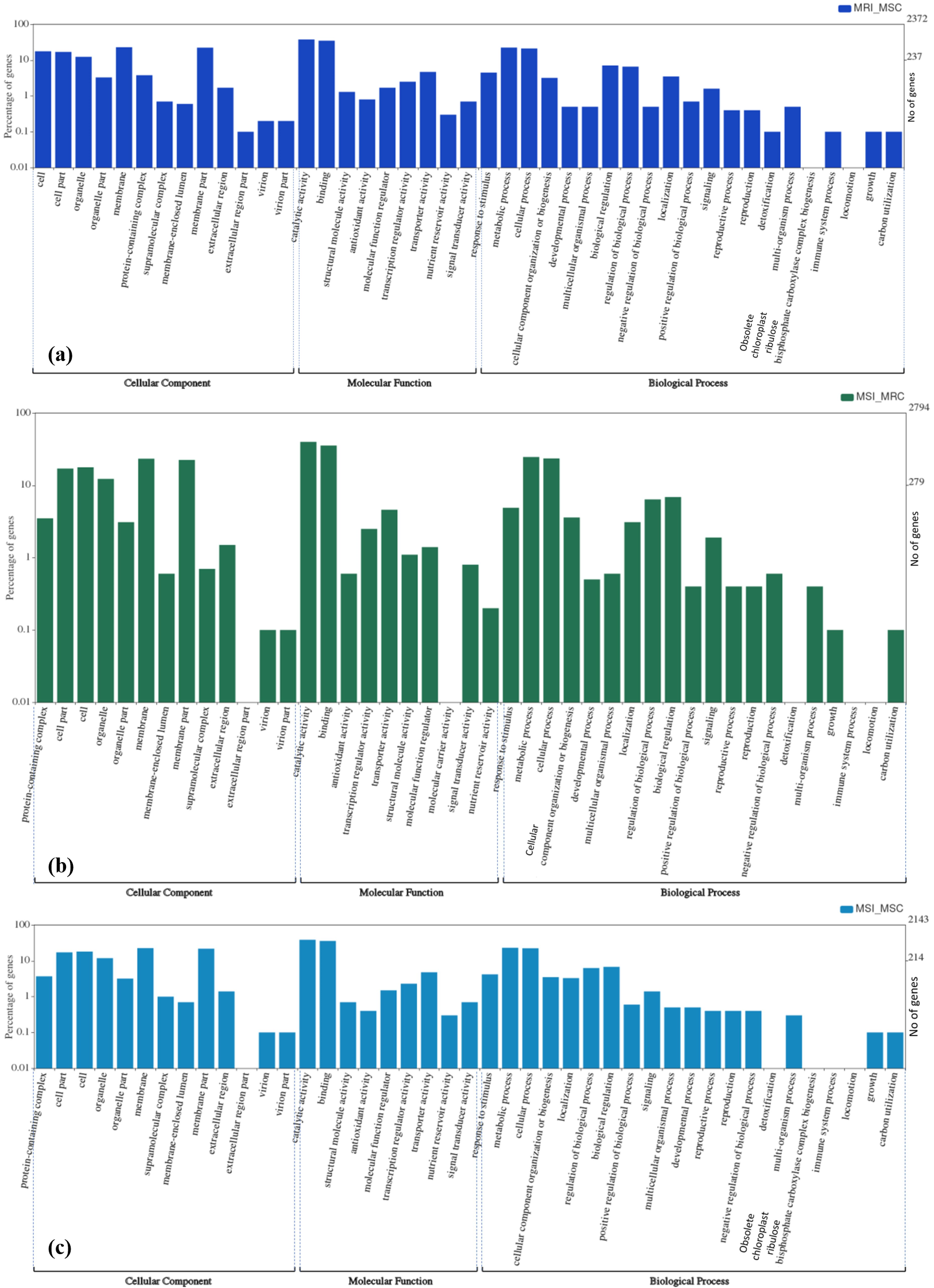

Supplement: S3 Fig — Gene ontology (GO) categorization of the differentially expressed genes (DEGs) in V. radiata in (a) Resistant Infected (MRI) vs Susceptible Control (MSC), (b) Susceptible Infected (MSI) vs Resistant Control (MRC), (c) Susceptible Infected (MSI) vs Susceptible Control (MSC), combinations. There are three main categories of biological process, cellular component and molecular function. Where Y-axis represents the unigene percentage. (TIF) [file pone.0244593.s003.tif]

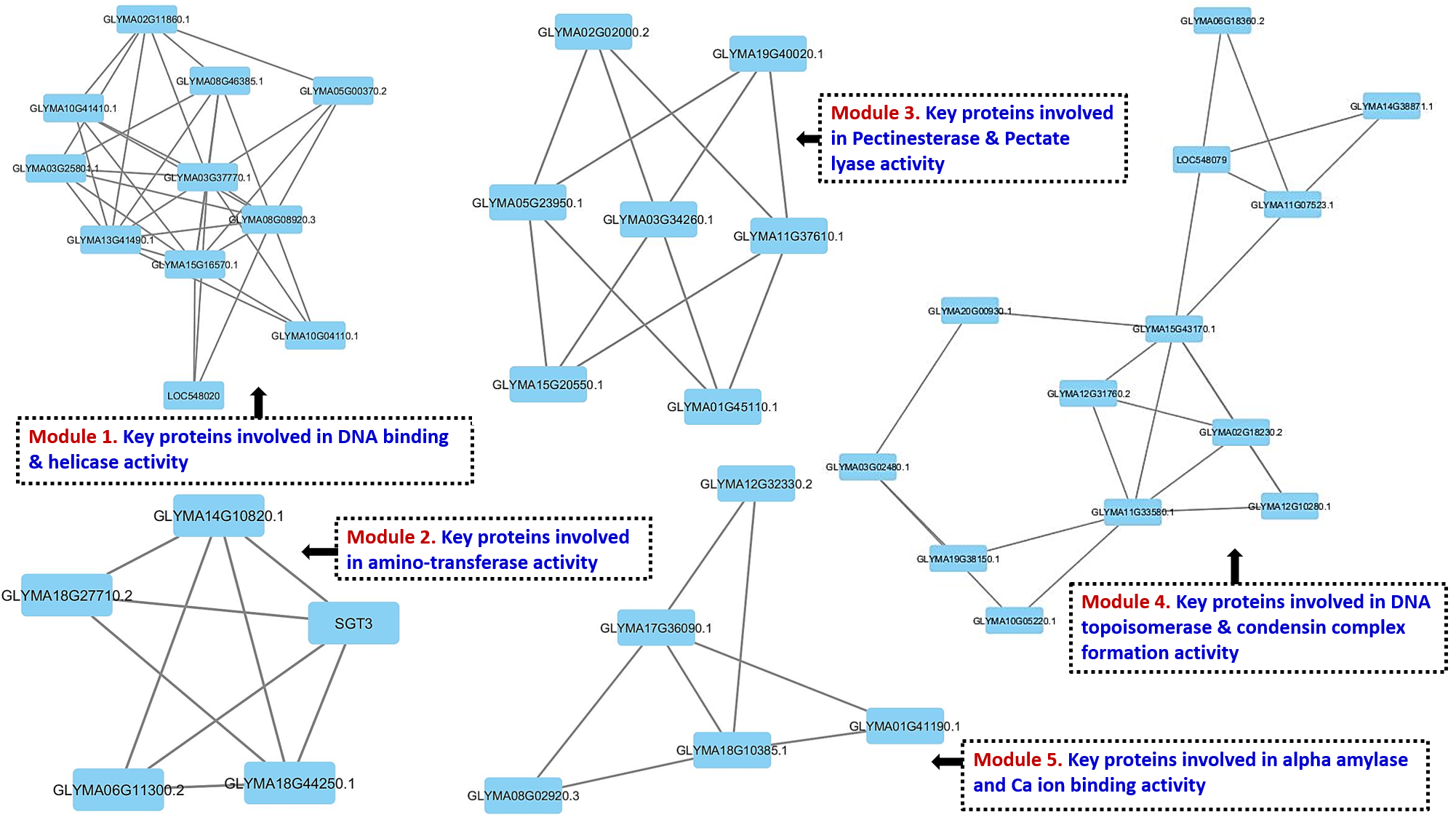

Supplement: S4 Fig — (TIF) [file pone.0244593.s004.tif]

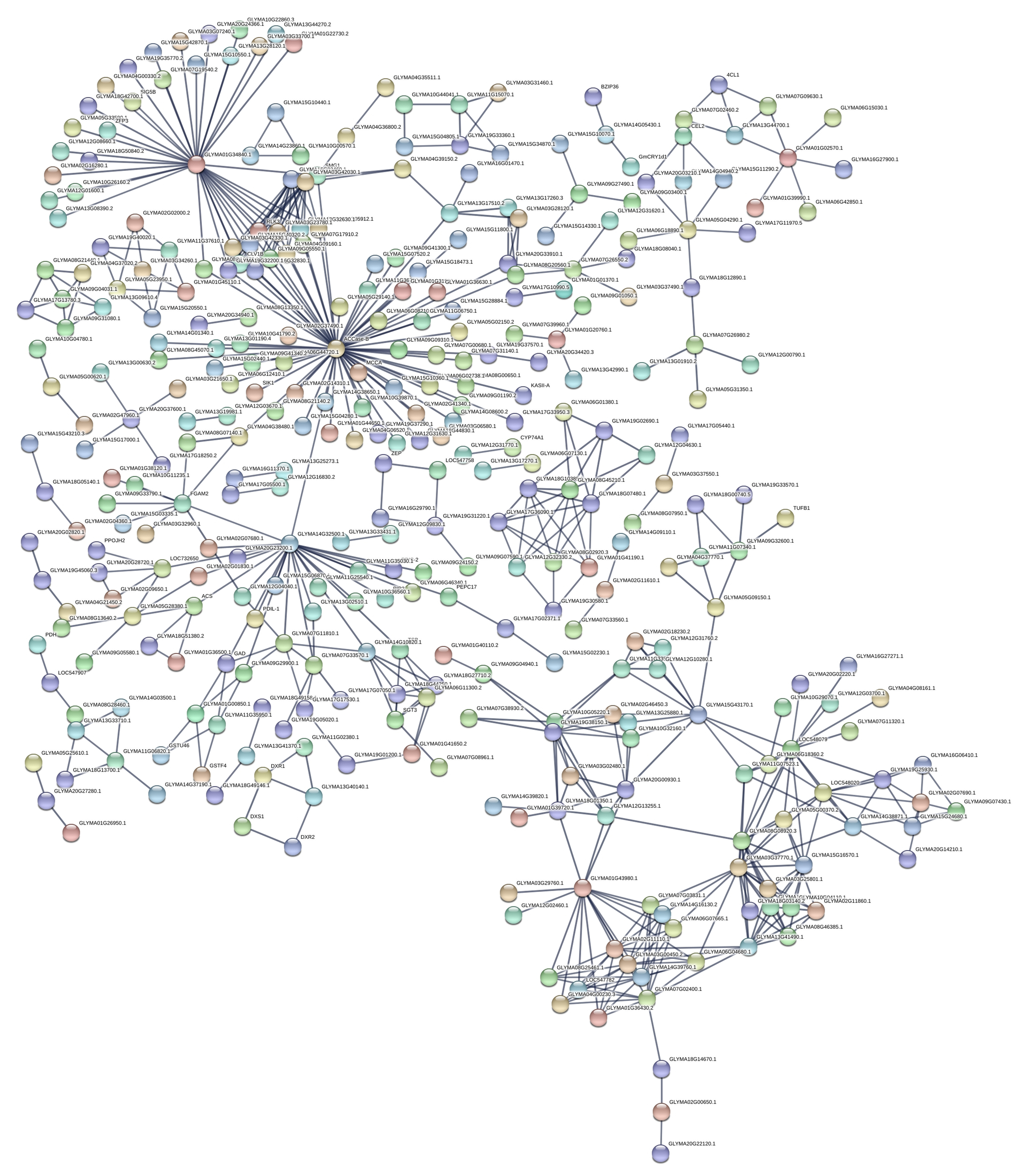

Supplement: S5 Fig — (TIF) [file pone.0244593.s005.tif]

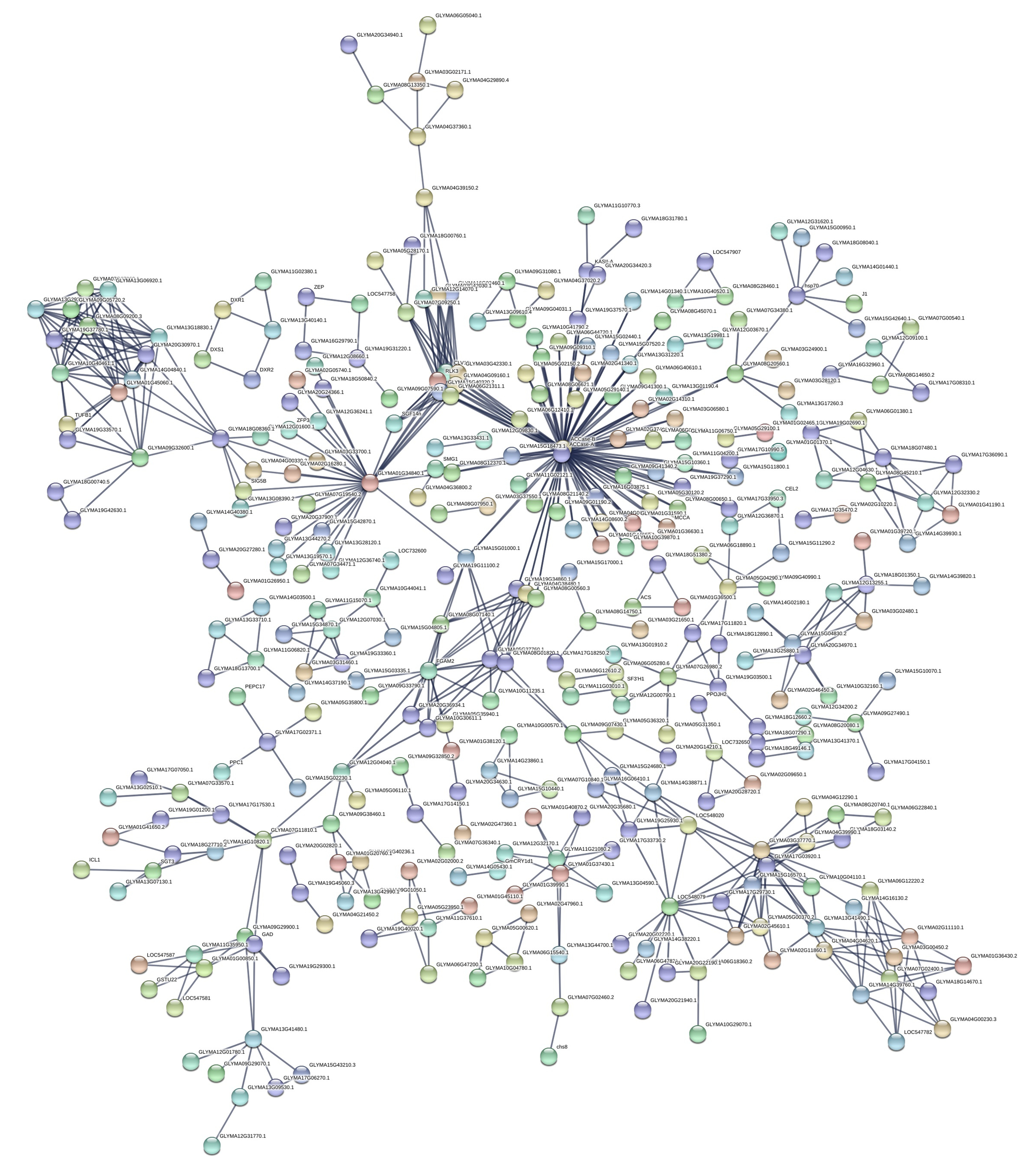

Supplement: S6 Fig — (TIF) [file pone.0244593.s006.tif]

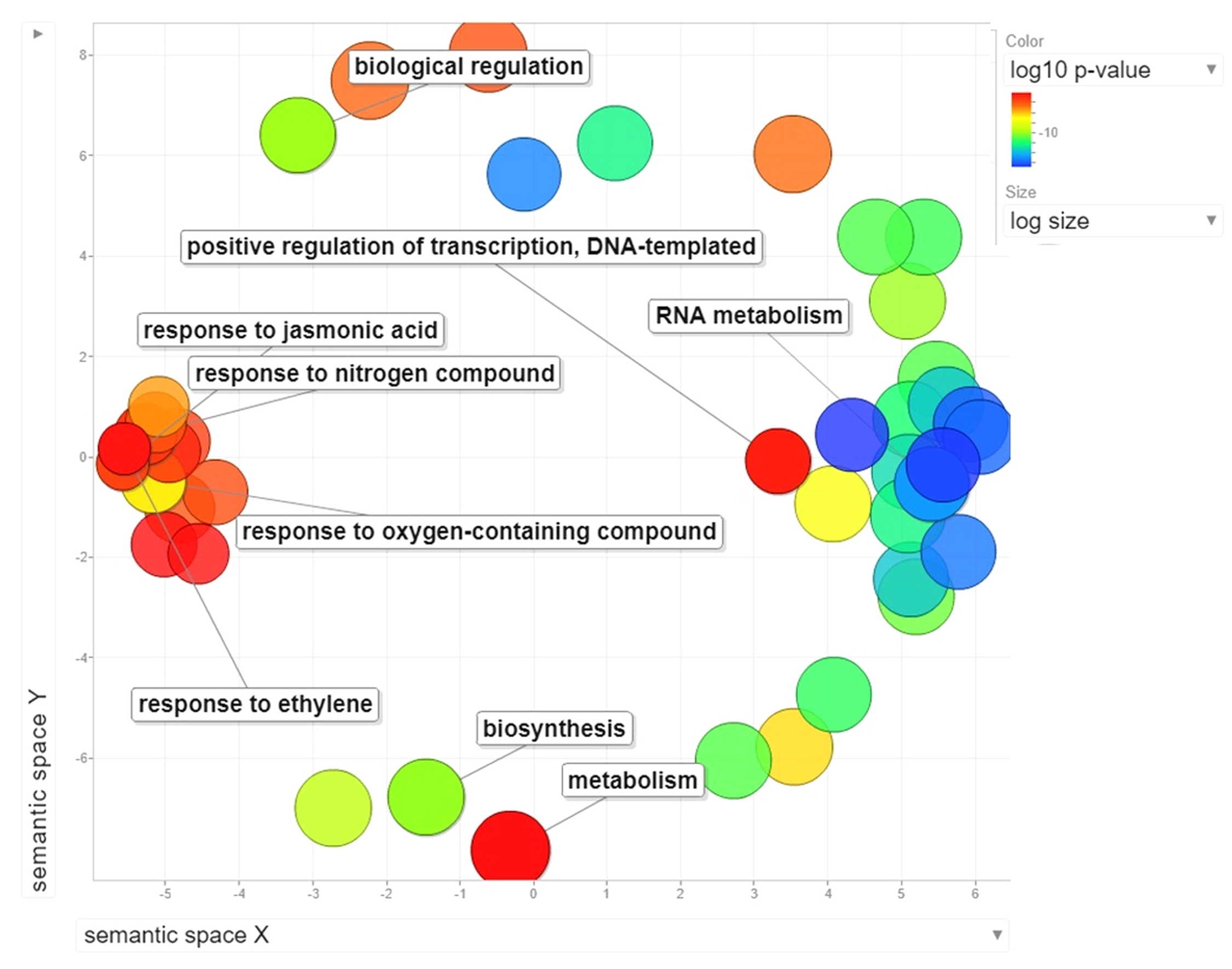

Supplement: S7 Fig — (TIF) [file pone.0244593.s007.tif]
