# Supplementary material for: Comparative RNA-Seq analysis unfolds a complex regulatory network imparting yellow mosaic disease resistance in mungbean [Vigna radiata (L.) R. Wilczek]
Source: PLoS One. 2021 Jan 12;16(1):e0244593. doi: 10.1371/journal.pone.0244593 (PMC7802970; doi:10.1371/journal.pone.0244593)
Supplement: S1 File — (PDF) [file pone.0244593.s008.pdf]

Supplementary file 1

MYMIV inoculation procedure

To confirm the resistance of PMR-1, whitefly transmission was carried out under controlled condition with standard procedure (Bag et al., 2014). Briefly, five healthy whiteflies maintained in *Nicotiana tabacum* was allowed to feed on the virus source plant as mentioned in the main text for 24 h to acquire the virus and released to individual PMR-1 and Pusa Vishal plants (total 10 plants each were inoculated, each with 5 whitefly) with inoculation access period of 24 h. The plants used for the infection were grown under control conditions and were nearly 20-25 days old. After killing the whiteflies with a systemic insecticide, the inoculated plants were maintained in cages to monitor the symptoms. The samples were collected for RNA Seq analysis after 7 days of inoculation. The yellow mosaic symptom appeared only on the susceptible (Pusa Vishal) genotype while no symptom was recorded on the resistant (PMR-1) genotype (Fig. 1).

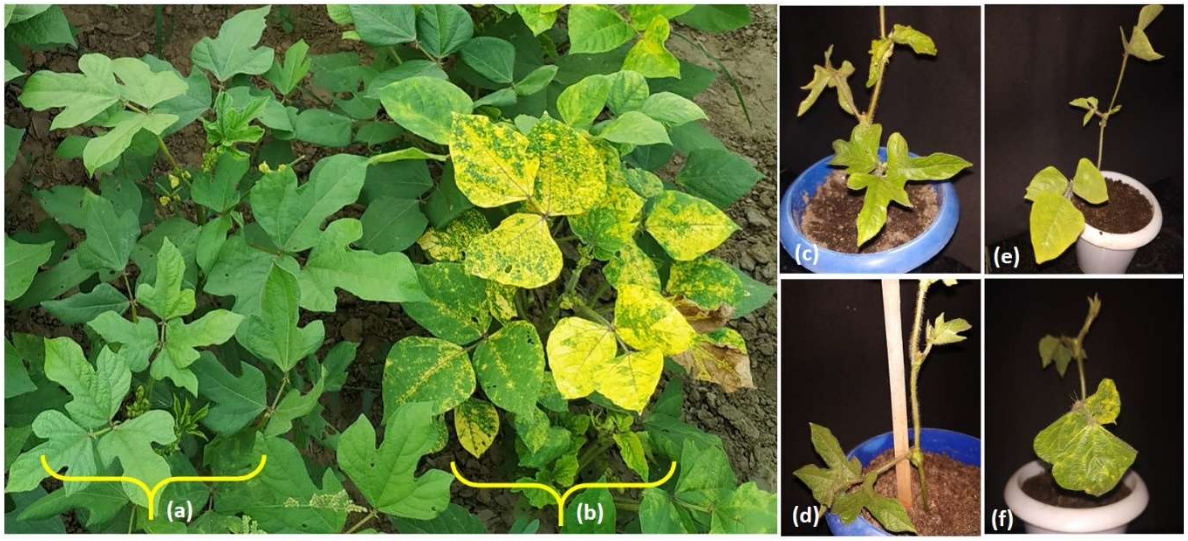

Fig. 1. MYMIV infection under open-field and under challenge inoculated conditions. Where, (a) PMR-1 and (b) Pusa Vishal under open filed conditions; (c) PMR-1 control; (d) PMR-1 inoculated; (e) Pusa Vishal control; (f) Pusa Vishal inoculated.

Confirmation of YMV infection through RCA and MYMIV and MYMV specific primers

Samples of symptomatic mungbean leaves were collected from the experimental field of IARI and presence of begomovirus was detected using pair MYMIV and MYMV specific primers. The virus was found to be a strain of MYMIV. One of the infected plants in which the MYMIV was detected, was used as an initial source of inoculum for this study. Total DNA was isolated from the tip region of the inoculated leaves (both PMR-1 and Pusa Vishal) and viral DNA copy number was enriched by polymerase chain reaction (PCR) (Saiki et al., 1988) using phi29 DNA polymerase-mediated rolling circle amplification (RCA) through the use of random primers (Dean et al., 2001). The RCA product from susceptible genotypes were eluted and digested using BamHI, as no amplification was observed for the resistant genotype (PMR-1) (Fig. 2).

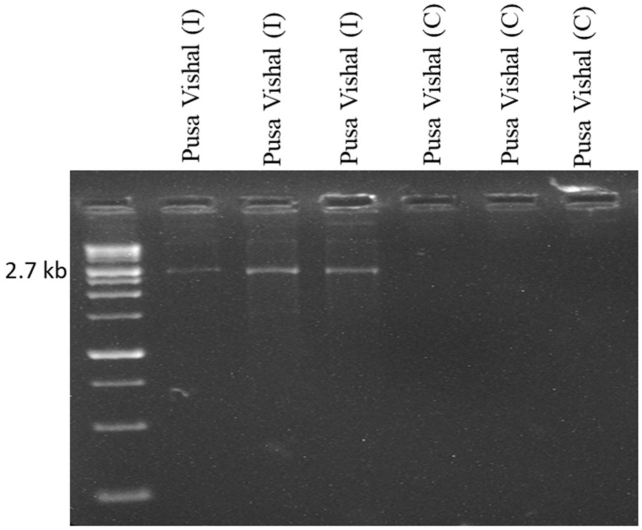

Fig. 2. Representative gel picture showing digested RCA product from susceptible mungbean genotype (Pusa Vishal) under infected (I) and control (C) conditions showing 2.7 kb fragment. (Resistant genotype could not amplify any product in RCA)

Further, the amplified product was diluted @1:20 (DNA:Water) and used for the detection of virus(es). For the identification of MYMIV, AV1 gene specific primers (MYMIV-Fwd: 5'GGAGGTGTCCCTACCAACATG3' and Rev: 5'CGATTCACCATGGCTTGTTCC3') were used having expected amplification size of ~500 bp; while for MYMV identification the AV1 (forward) and AC2 (reverse) gene specific primers (MYMV-Fwd: 5'GGAAGTGTCCCTGCCAGCG3' and Rev: 5'CCACAGGTTGAAGAAAGCAC3') were used having expected amplification size of ~925 bp, as per the below mentioned protocol. MYMIV specific amplification (~500 bp) was observed in case of susceptible genotype Pusa Vishal under infected condition while no amplification could be recorded for the resistant genotype (PMR-1) under infected or control conditions, or for susceptible genotype (Pusa Vishal) under control condition (Fig. 3).

PCR reaction mixtures and Program of primers of MYMIV and MYMV

PCR reaction mixture: Total volume 25 µL

10X Buffer-	2.5 µl
dNTP-	0.5 µl
FP-	0.5 µl
RP-	0.5 µl
Taq Enzyme-	1.0 unit
Template-	400-500 ng (1 µl)
Water-	20 µl
Total-	25 µl

Note: FP- Forward primer; RP- Reverse primer. The above PCR reaction mixture is applicable for all the pairs of primers

AV1 gene specific primers for MYMIV

PCR program of MYMIV-Fwd & Rev (Amplicon size ~500 bp)

Preheating temp-	94°C- 2 to 3 minutes
Denaturation-	94°C- 30 Second
Annealing temp.-	56°C- 45 second
Extension-	72°C- 40 second (in normal taq enzyme)
Final extension-	72°C- 10 minutes
	04°C- Infinite

AV1 (forward) and AC2 (reverse) gene specific primers for MYMV
PCR program for MYMV-Fwd & Rev (Amplicon size ~925bp)

Preheating temp-	94°C- 2 to 3 minutes
Denaturation-	94°C- 30 Second
Annealing temp.-	60°C- 45 second
Extension-	72°C- 60 second (in normal taq enzyme)
Final extension-	72°C- 10 minutes
	04°C- Infinite

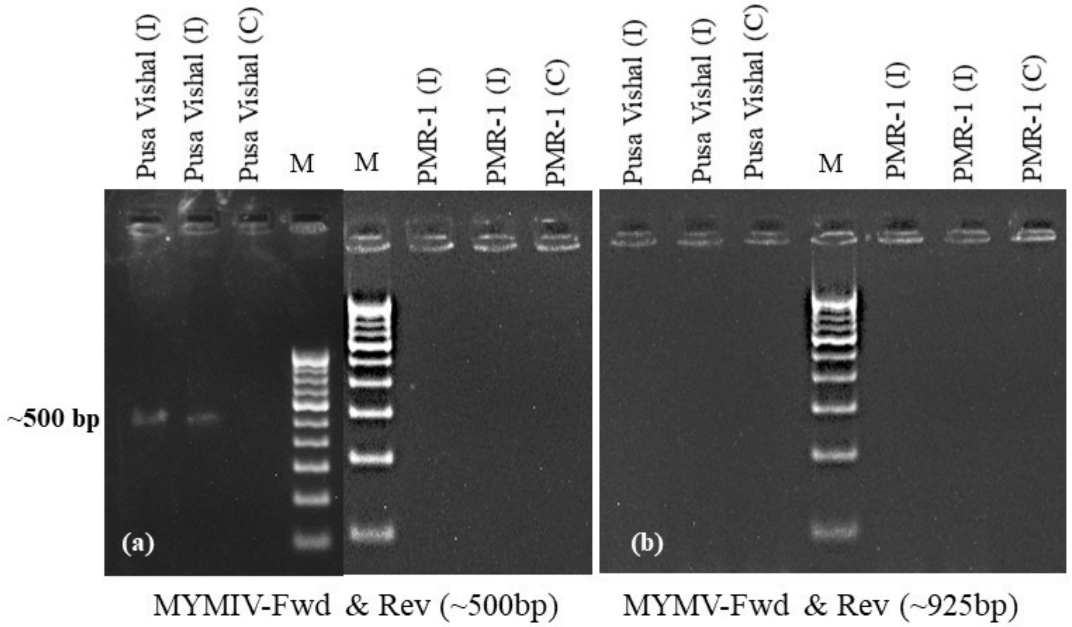

Fig. 3. Viral gene specific amplification under infected and control conditions of resistant and susceptible mungbean genotypes using (a) MYMIV and (b) MYMV specific primers.

(a) MYMIV specific amplification (~500 bp) in the susceptible genotype Pusa Vishal under infected conditions while no amplification could be recorded for the resistant genotype (PMR-1) under infected or control conditions or susceptible genotype (Pusa Vishal) under control conditions. (b) No amplification in either resistant or susceptible genotypes under infected or control conditions using MYMV (Fwd & Rev) specific primers. Where, M: DNA ladder; I: Inoculated sample; C: Control sample; two inoculated and one control plant was used for the primer amplification.

References

Bag MK, Gautam NK, Prasad TV, Pandey S, Dutta M, Roy A (2014) Evaluation of an Indian collection of black gram germplasm and identification of resistance sources to *Mungbean yellow mosaic virus. Crop Prot*. 61: 92-101. doi:10.1016/j.cropro.2014.03.021

Dean FB, Nelson JR, Giesler TL, Lasken RS (2001) Rapid amplification of plasmid and phage DNA using Phi 29 DNA polymerase and multiply-primed rolling circle amplification. *Genome Res.* 11:1095–1099.

Saiki RK, Gelfand DH, Stoffel S, Scharf SJ, Higuchi R, Horn GT, Mullis KB, Erlich HA (1988) Primer-directed enzymatic amplification of DNA with a thermostable DNA polymerase. *Science* 239:487–491.
