# Supplementary material for: Comparative RNA-Seq analysis unfolds a complex regulatory network imparting yellow mosaic disease resistance in mungbean [Vigna radiata (L.) R. Wilczek]
Source: PLoS One. 2021 Jan 12;16(1):e0244593. doi: 10.1371/journal.pone.0244593 (PMC7802970; doi:10.1371/journal.pone.0244593)
Supplement: S2 Table — (DOCX) [file pone.0244593.s010.docx]

**S2 Table. Alignment details of total paired and unpaired reads.**

| **A. Total paired reads** | **MRI** | **MSI** | **MRC** | **MSC** |
| --- | --- | --- | --- | --- |
| 1. Aligned concordantly or   discordantly 0 time: | 3127775  (17.77%) | 2538918  (12.83%) | 2118886  (11.00%) | 2121163  (12.80%) |
| 1. Aligned concordantly   1 time: | 13172080  (74.81%) | 16045044  (81.07%) | 15899619  (82.52%) | 13370106  (80.69%) |
| 1. Aligned concordantly   >1 times: | 832150  (4.73%) | 653148  (3.30%) | 671894  (3.49%) | 605423  (3.65%) |
| 1. Aligned discordantly   1 time: | 474372  (2.69%) | 554425  (2.80%) | 576907  (2.99%) | 473908  (2.86%) |
| **Total** | **176,06,377** | **197,91,535** | **192,67,306** | **165,70,600** |
| **B. Total unpaired reads:** |  |  |  |  |
| 1. Aligned 0 time: | 4656703  (74.44%) | 3283050  (64.65%) | 2560664  (60.42%) | 2731716  (64.39%) |
| 1. Aligned 1 time: | 1471393  (23.52%) | 1699559  (33.47%) | 1587736  (37.47%) | 1430344  (33.72%) |
| 1. Aligned >1 times: | 127454  (2.04%) | 95227  (1.88%) | 89372  (2.11%) | 80266  (1.89%) |
| **Total** | **62,55,550** | **50,77,836** | **42,37,772** | **42,42,326** |
| **C. Overall alignment rate:** | 86.78% | 91.71% | 93.35% | 91.76% |
| **D. Other details** |  |  |  |  |
| i. PE one mate mapped uniquely | 735696.5 | 849779.5 | 793868.0 | 715172.0 |
| ii. PE neither mate aligned | 2328351.5 | 1641525.0 | 1280332.0 | 1365858.0 |
| iii. PE one mate multi-mapped | 63727.0 | 47613.5 | 44686.0 | 40133.0 |
